# Programmable and robust static topological solitons in mechanical metamaterials

**DOI:** 10.1038/s41467-019-13546-y

**Published:** 2019-12-06

**Authors:** Yafei Zhang, Bo Li, Q. S. Zheng, Guy M. Genin, C. Q. Chen

**Affiliations:** 1 Department of Engineering Mechanics, CNMM and AML, Tsinghua University, 100084 Beijing, P.R. China; 2 Mechanical Engineering and Materials Science, Washington University, St. Louis, MO 63130 USA; 3 NSF Science and Technology Center for Engineering Mechanobiology, St. Louis, MO 63130 USA

**Keywords:** Phase transitions and critical phenomena, Metamaterials, Topological defects

## Abstract

Solitary, persistent wave packets called solitons hold potential to transfer information and energy across a wide range of spatial and temporal scales in physical, chemical, and biological systems. Mechanical solitons characteristically emerge either as a single wave packet or uncorrelated propagating topological entities through space and/or time, but these are notoriously difficult to control. Here, we report a theoretical framework for programming static periodic topological solitons into a metamaterial, and demonstrate its implementation in real metamaterials computationally and experimentally. The solitons are excited by deformation localizations under quasi-static compression, and arise from buckling-induced kink-antikink bands that provide domain separation barriers. The soliton number and wavelength demonstrate a previously unreported size-dependence, due to intrinsic length scales. We identify that these unanticipated solitons stem from displacive phase transitions with periodic topological excitations captured by the well-known ${\varphi }^{4}$ theory. Results reveal pathways for robust regularizations of stochastic responses of metamaterials.

## Abstract (web summary)

Mechanical solitons are notoriously difficult to control. Here, the authors report a theoretical framework for programming static periodic topological solitons into a metamaterial, and demonstrate its implementation in real metamaterials computationally and experimentally.

## Introduction

The localized deformation underlying kink-excitations drives numerous nonlinear physical and mechanical phenomena including fault zones in earthquake^1^, shear bands in granular materials^2^, folds of soft systems^3–5^, as well as geometric phase transitions in metamaterials^6–8^. Localizations associated with solitons explain many fundamental features of dislocations, ferroelectric domain walls, surface diffusion, conducting polymers, intrinsic localized modes, and nonlinear excitations in biological molecules^9–12^. Although they have been observed in solitary excitations of mechanical metamaterials^13,14^ and domain-wall formations of van der Waals layered materials^15–17^, they have previously been stochastic and scale-independent discrete and/or single-soliton systems. A longstanding goal has been development of programmable, localized topological structures to control solitons^18^. This technology would hold prospects not only in tunable photonic and phononic rectification^19^, but also in designing smart soft robots and high sensitivity devices^20^.

Extensive work has been focused not only on engineering localizations into civil structures (e.g. crash bands, shear bands and plastic twinning)^21,22^, but also on designing desired patterns and interfaces in architected metamaterials^6,8,23,24^. The central challenge is the widely-recognized problem that localized deformations are sensitive to inherent or externally induced defects, rendering the location and dimension of localization zones hard to predict^6,23,25,26^. Successful previous efforts include ordered configurations achieved by introducing long-range interactions^27^, geometric frustration^28^, periodic dopants or macro-defects^29^. However, generation of programmable, ordered and robust static solitons and ensuing localized instabilities in nominally defect-free mechanical systems still remains largely unexplored.

Inspired by recent numerical work indicating that excitations of ordered localizations can be achieved via carefully designed architectures^30^, here we develop a general framework for analyzing this entire class of materials through the formalism of static periodic solitons. Many have previously addressed the bi-stability of metamaterials through conventional snap-though equilibrium techniques^6,8,23,24,31,32^. The general framework presented here is based on an approach to the underlying physics whereby interactions between double-well on-site potentials of the unit cell and the strong coupling energies of adjacent unit cells determine how metamaterial architecture and loading produce buckling-induced kink and antikink deformation bands that provide domain separation barriers.

We therefore explored the instability of cellular metamaterials subjected to quasi-static compression. We found that, beyond a critical loading, deformation symmetry breaks down, leading to two distinct macroscopic buckling modes separated by localized topological interfaces that are static periodic solitons (kink-antikink pairs) governed by the well-known ${\varphi }^{4}$ equation^33,34^. This unanticipated behavior originates from a structural phase transition analogous to mechanical soliton-lattices of magnetic systems^11,35^. We theoretically illustrate that the period of the soliton-lattice in fact introduces an intrinsic length scale perpendicular to the compression direction, which leads to novel size-dependence of the soliton number and wavelength in the metamaterial.

## Results

### Metamaterial and static solitons

**Fig. 1:**
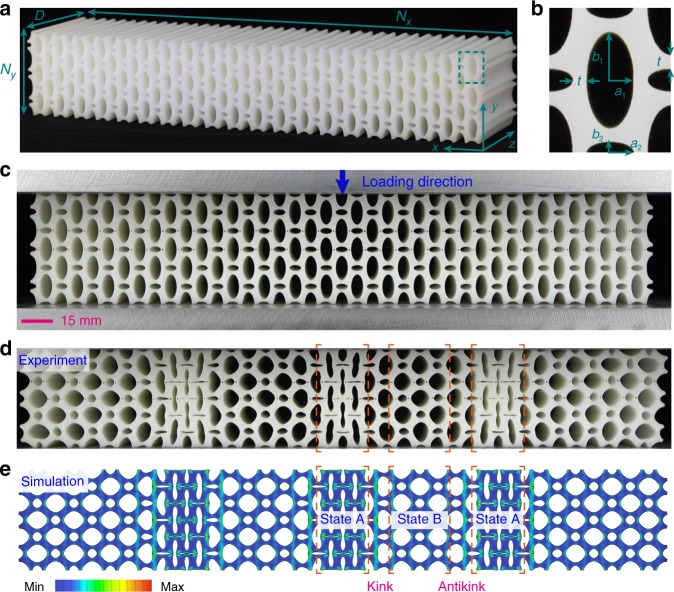
Structural phase transitions in a mechanical metamaterial lead to static soliton formation. **a** A rubber metamaterial patterned with a regular array of elliptic holes. **b** The geometric parameters of the unit cell, here characterized by elliptical-axes ${a}_{1}={a}_{2}=$ 3 mm and ${b}_{1}=4{b}_{2}=$ 6 mm, and by neck thickness $t=$ 1.5 mm. **c** Static solitons were activated by compression between two lubricated, parallel plates. **d**, **e** Uniform compression induces symmetry breaking, with two distinct buckling states A and B emerging in the experimental (**d**) and numerical (**e**) models. Colors in **e** illustrate the simulated von Mises stress field in the deformed configuration. Static periodic solitons (alternating kinks and antikinks) are localized at the domain walls between two uniform polarization states.

We demonstrated these in a metamaterial consisting of a periodic array of alternate through-thickness elliptic holes of dual sizes (cf. Fig. 1a for a sample comprising ${N}_{x}\times {N}_{y}=20\, \times 3$ unit cells) in a coated elastomer (see Methods and Supplementary Fig. 1 for details). Uniaxial compression applied to two parallel lubricated plates (Fig. 1c) was performed at a sufficiently low strain rate ($3.1\, \times 1{0}^{-5}\ {{\rm{s}}}^{-1}$) to mimic quasi-static loading; although elastomers are in general viscoelastic, this loading rate was deemed sufficiently slow to mimic quasi-static loading because no memory effects were evident in metamaterial samples under three consecutive loadings. Under small compressive strains $\varepsilon =2\delta /{Y}_{0}$, where $2\delta$ is the vertical displacement and ${Y}_{0}$ is the sample height, the sample underwent affine deformation and maintained its initial symmetry group ${D}_{2}\times {S}_{N}$ (the direct product of the reflection symmetries of the rectangle, ${D}_{2}$, and all permutations of the $N={N}_{x}\times {N}_{y}$ unit cells, ${S}_{N}$). When the compressive strain exceeded a threshold strain ${\varepsilon }_{{\rm{c}}}$, the vertical ligaments within unit cells buckled and the compressive load deviated from the linear response and then decreased. Spontaneous symmetry breaking took place, leading to a striking pattern with lower symmetry (i.e., breaking the original permutation symmetries of unit cells) and two alternate macroscopic buckling states (Fig. 1d, e at $\varepsilon =10.7 \%$). The deformation pattern consisted of ordered elastic instabilities analogous to displacive phase transitions in ferroelectric systems^36,37^. Between these two states, highly localized topological domain walls emerged to accommodate the incompatible deformation (see Supplementary Movie 1). Although deformation localization observed in other systems can be stochastic^7,15,17,23,25,26^, the localization reported here are ordered and robust (as confirmed by 6 tests for each case). To verify the structural origin of this order, we performed 2D plane strain finite element (FE) simulations that reproduced experimental observations (see Fig. 1, Supplementary Fig. 2).

### Physical model and periodic-soliton solution

**Fig. 2:**
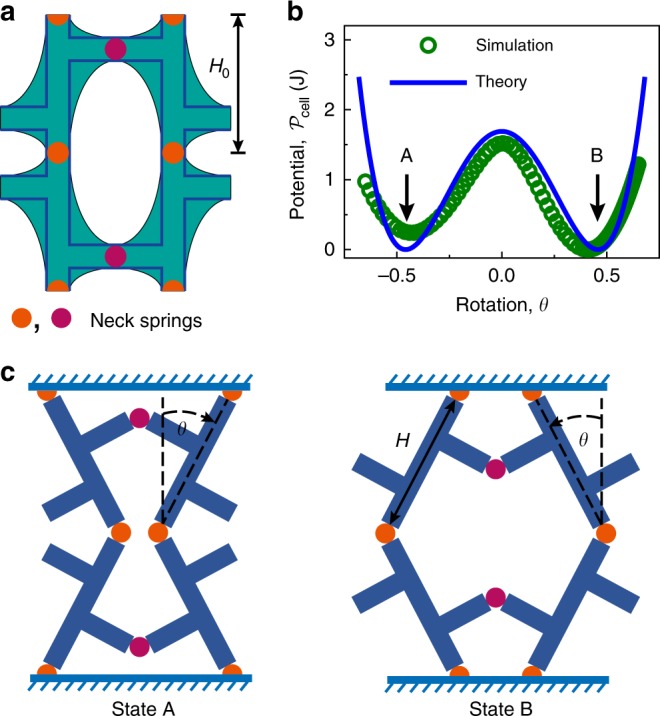
Bi-stability of the unit cell under compression. **a** Schematic of the unit cell and the corresponding theoretical model. The simplified theoretical model consisting of elastic rods and neck springs (yellow and magenta dots) is illustratively superimposed on the unit cell of the sample. **b** The on-site potential ${{\mathcal{P}}}_{{\rm{cell}}}\left(\delta ;\theta \right)$ of the unit cell under vertical compression strain $\varepsilon =10.7 \%$. **c** Two possible buckling configurations (polarization states) of the unit cell corresponding to the two equilibrium states of the double-well potential shown in **b**.

To identify the physical underpinnings of these phenomena, the unit cell shown in Fig. 1b was simplified as a structure made of rods and neck springs (rod-spring model in Fig. 2a). When designing the mechanical metamaterial, we specially choose ${a}_{1}={a}_{2}$ and the ligament width between two ellipses (i.e., cell neck thickness) $t$ to be the same. By doing so, all the vertical rods are collinear to ensure the transition of deformation from stretching to bending-dominated during buckling. Both experimental and FE numerical results show that the buckling deformation of the metamaterial is mainly localized in the narrow regions of slender necks (marked by dots in Fig. 2 and Supplementary Fig. 3). Therefore, the connection between the elastic rods can be approximated by springs located at the necks, with the torsional stiffness denoted by ${C}_{1}$ and ${C}_{2}$ for the vertical and horizontal rods. Two symmetric modes of the simplified model exist and can be characterized by the rotation angle $\theta$ of the vertical rods (Fig. 2b, c). We adopt the notion of polarization in structural phase transitions to metamaterials by introducing the inward polarization for $\theta \, < \,0$ and outward polarization for $\theta \, > \, 0$ of the unit cell corresponding to the states A and B (Figs. 1b and 2c), respectively. Here, $\theta$ is the order parameter in structural phase transitions. When the compressive strain $\varepsilon$ approaches the critical value ${\varepsilon }_{{\rm{c}}}$ (i.e., $\varepsilon \to {\varepsilon }_{{\rm{c}}}$), the angle $\theta$ bifurcates into two opposite polarizations, breaking the initial symmetry of the structure.

The elastic strain energy of the simplified bar model is given by ${U}_{{\rm{cell}}}\left(\delta \right)=2(2{C}_{1}+{C}_{2}){\theta }^{2}+2K{(\Delta H)}^{2}$, where $\Delta H={H}_{0}-({H}_{0}-\delta )\sec \theta$ is the shortening of the vertical bars and $K$ is the effective stiffness of the stretching springs that mimic the vertical bars. As $\theta \ll 1$, the strain energy can be approximately rewritten as a polynomial form ${U}_{{\rm{cell}}}\left(\delta \right)={U}_{e}{({\theta }^{2}-{\Theta }_{{\rm{cell}}})}^{2}+{D}_{1}$ with ${U}_{e}=K(3{H}_{0}-8\delta )({H}_{0}-\delta )/6$, ${\Theta }_{{\rm{cell}}}=\left(K\delta ({H}_{0}-\delta )-(2{C}_{1}+{C}_{2})\right)/{U}_{e}$ and ${D}_{1}=2K{\delta }^{2}-{\Theta }_{{\rm{cell}}}^{2}{U}_{e}$ (see Supplementary Note 3). Here, we consider the unit cell as a ‘quasiparticle’ and introduce an on-site potential ${{\mathcal{P}}}_{{\rm{cell}}}\left(\delta ;\theta \right) \,\, \triangleq \,\, {U}_{e}{({\theta }^{2}-{\Theta }_{{\rm{cell}}})}^{2}$, in which the unit cell admits its actual configuration at the well bottoms. The potential energy ${{\mathcal{P}}}_{{\rm{cell}}}$ at a selected compressive strain of $10.7 \%$ obtained from FE simulation is shown in Fig. 2b, in comparison with the corresponding theoretical prediction given by $K$ = 1.478 N mm${}^{-1}$, ${C}_{1}=$ 1.150 N mm and ${C}_{2}$ = 1.069 N mm (see Supplementary Fig. 3). Our simulations show that, at small compressive strains (i.e., $\varepsilon \ll {\varepsilon }_{{\rm{cell}}}$), the potential ${{\mathcal{P}}}_{{\rm{cell}}}\left(\delta ;\theta \right)$ has only one stable state, while at large strains (i.e., $\varepsilon \, > \, {\varepsilon }_{{\rm{cell}}}$) it switches from mono-stable to bi-stable (see Supplementary Fig. 7e). This switch is attributed to the deformation transition of the unit cell from stretching to bending at ${\varepsilon }_{{\rm{cell}}}=\left(1-\sqrt{1-4(2{C}_{1}+{C}_{2})/(K{H}_{0}^{2})}\right)/2$ which can be deduced from losing convex in ${{\mathcal{P}}}_{{\rm{cell}}}$. The two stable states in the bi-stable potential correspond to polarization A ($\theta \, < \, 0$) and B ($\theta \, > \, 0$), respectively.

**Fig. 3:**
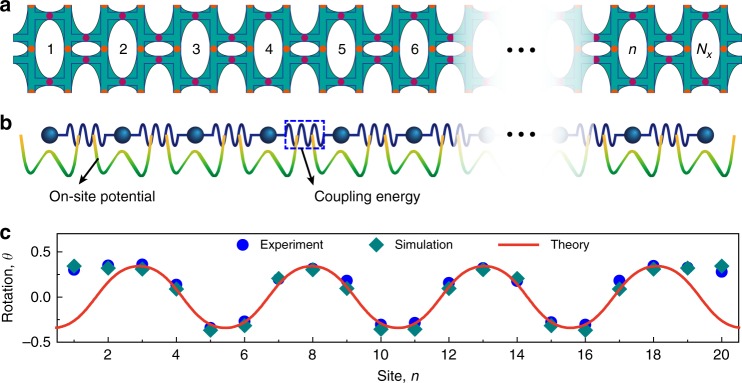
One-dimensional model and the topological periodic-soliton solution. **a** One-dimensional mechanism model, the length of which underlies the design of the 1D cellular metamaterial chain. **b** A 1D particle chain interacting with a double-well potential serves as an equivalent, general physical model to reveal the formations of topological defects. The particle and coupling energy represent the unit cell, and the shear and bending between nearest cells, respectively. **c** The experimental and simulated rotation field for a $20\, \times 3$ specimen at a strain $\varepsilon =10.7 \%$ is predicted by the ${\varphi }^{4}$ topological periodic-soliton solution.

Both experiments and simulations show that the macroscopic buckling and localized deformation in the metamaterial seem to be insensitive to the number of unit cells in the vertical direction (see Supplementary Figs. 2 and 4). Therefore, we can apply a one-dimensional (1D) model constructed by a chain of simplified unit cells (Fig. 3a) to uncover the deformation mechanism of the metamaterial shown in Fig. 1d. The Lagrangian ${\mathcal{L}}$ of the discrete 1D metamaterial chain consists of the sum of the on-site potential and the coupling energy between the adjacent unit cells (Fig. 3b). Denoting the characteristic angle of the $n$-th unit cell by ${\theta }_{n}$ (see Supplementary Fig. 2 and Note 3), one has

1 $${\mathcal{L}}={\sum _{n}}{C}_{{\rm{s}}}{s}_{n-1,n}^{2}+2{C}_{2}{\theta }_{n}^{2}+{U}_{{\rm{cell}}}\left({\theta }_{n}\right)$$

where ${s}_{n-1,n} \,\, \triangleq \,\, d(\sin {\theta }_{n-1}-\sin {\theta }_{n})$ and ${C}_{{\rm{s}}}$ are the shear deformation and stiffness between the $(n-1)$-th and $n$-th cell, respectively. Adjacent unit cells are thus coupled by shear and bending deformations represented by the first two terms in Eq. (1), and the former also denotes the interaction energy between nearest-neighbor cells. Under the condition $\theta \ll 1$, a set of equilibrium equations can be obtained from the Lagrangian as

2 $$-2{C}_{{\rm{s}}}{d}^{2}({\theta }_{n-1}+{\theta }_{n+1}-2{\theta }_{n})+{\partial }_{{\theta }_{n}}{{\mathcal{P}}}_{{\rm{eff}}}\left({\theta }_{n}\right)=0$$

where ${{\mathcal{P}}}_{{\rm{eff}}}\left({\theta }_{n}\right)={U}_{e}{({\theta }_{n}^{2}-{\Theta }_{{\rm{eff}}})}^{2}$ is the effective on-site potential, with ${\Theta }_{{\rm{eff}}}=(K\delta ({H}_{0}-\delta )-2({C}_{1}+{C}_{2}))/{U}_{e}$. It is clear that when ${\Theta }_{{\rm{eff}}}\, > \,0$, ${{\mathcal{P}}}_{{\rm{eff}}}\left({\theta }_{n}\right)$ is a double-well potential with two minima at ${\theta }_{n}=\pm \sqrt{{\Theta }_{{\rm{eff}}}}$; when ${\Theta }_{{\rm{eff}}}\, < \, 0$, on the contrary, ${{\mathcal{P}}}_{{\rm{eff}}}\left({\theta }_{n}\right)$ only has one stable state and the system undergoes uniform deformations under compression. We next examine the former case where two limiting physical regimes such as the order-disorder and displacive phase transitions can be expected (Supplementary Note 3). Essentially, these transitions depend on the relative strength of the interaction energy $4{C}_{{\rm{s}}}{d}^{2}{\Theta }_{{\rm{eff}}}$ and the effective on-site potential barrier ${U}_{e}{\Theta }_{{\rm{eff}}}^{2}$^11,36^. We further focus on the strong coupling $4{C}_{{\rm{s}}}{d}^{2}\gg {U}_{e}{\Theta }_{{\rm{eff}}}$ (i.e., ${C}_{{\rm{s}}}\gg (K{H}_{0}^{2}-8({C}_{1}+{C}_{2}))/16$) and consider the displacive case of Eq. (2) with a double-well on-site potential. To get the continuum limit (model), we define a slowly varying rotation field $\theta \left(x\right)$ by letting ${\theta }_{n}\to \theta \left(x=n\ell \right)$ and ${\theta }_{n\pm 1}\to \theta \left(n\ell \right)\pm \ell {\partial }_{x}\theta \left(n\ell \right)+{\ell }^{2}{\partial }_{xx}\theta \left(n\ell \right)/2$ in the discrete system (2), where $\ell =2({a}_{1}+{a}_{2}+t)$ is the lattice constant of the 1D metamaterial shown in Fig. 3. Accordingly, one obtains

3 $$\frac{{\partial }^{2}\theta }{\partial {x}^{2}}-\frac{\partial V\left(\theta \right)}{\partial \theta }=0$$

where $V\left(\theta \right)=\lambda {({\theta }^{2}-{\Theta }_{{\rm{eff}}})}^{2}/4$ and $\lambda =2{U}_{e}/({C}_{{\rm{s}}}{d}^{2}{\ell }^{2})$. Equation (3) is the well-known $\lambda {\varphi }^{4}$ field equation in structural phase transition^10,37^. For a finite-size superlattice with periodic boundary conditions, Eq. (3) permits the following nonlinear periodic solution with modulus $m$^37^:

4 $$\theta =a\cdot {\rm{sn}}\left[b\sqrt{\lambda /2}x;m\right]$$

where ${\rm{sn}}\left[x;m\right]$ is the Jacobi elliptic function, $a=\sqrt{2m/(1+m){\Theta }_{{\rm{eff}}}}$, $b=\sqrt{2/(1+m){\Theta }_{{\rm{eff}}}}$, and $m$ is determined by boundary conditions. Note that solution (4) represents a static topological periodic-soliton in the form of kink and antikink alternatively distributed in the system. The normalized period is $T=4{\rm{K}}\left[m\right]/(b\ell \sqrt{\lambda /2})$, where ${\rm{K}}\left[m\right]$ is the complete elliptic integral of the first kind. For a finite system, the resulted configuration is usually termed as a soliton-lattice with kink width $w=\sqrt{(1+m)/(\lambda {\Theta }_{{\rm{eff}}})}$^36^. It is also noted that all the involved spring stiffness and thus the soliton period can be substantively programmed by the geometry of the unit cells^30^. Moreover, the topological soliton solution (4) breaks the $\left(\theta ,x\right)\to \left(\theta ,-x\right)$ symmetry of the ordinary ${\varphi }^{4}$ equation and periodically separates two kinds of polarization domains which are degenerate ground states of the system.

We measure the characteristic angle $\theta$ of the samples in experiments and simulations by calculating the averaged relative rotations of the point pairs, marked on the vertical necks of each cell in the middle row (see Supplementary Fig. 2). The results of a sample with $20 \, \times 3$ unit cells at $\varepsilon =10.7 \%$ are plotted in Fig. 3c. As expected, both experimental and simulation results show that, characterized by the rotation angle $\theta$, the metamaterial is transformed into domains of uniform polarization, separated by narrow domain walls across which the polarization varies from one kind of orientation to another. The ordered configuration of domain walls features static periodic solitons which are pinned by the Perierls-Nabarro barrier^38^. The slight difference between the widths of states A and B is attributed to the actual asymmetric on-site potential energy of the unit cell (Figs. 2b and 3b), resulting from the finite thickness and irregular cross-sections of the neck regions. For comparison, our theoretical, experimental, and numerical results are shown in Fig. 3c, with a kink at ${x}_{0}=(T/4+21/2)\ell$ by setting $\theta =a\cdot {\rm{sn}}[b\sqrt{\lambda /2}(x-{x}_{0});m]$ and $T=5$. It can be seen that the theoretical soliton solution (4) agrees well with the experimental and numerical results, demonstrating that the stable topological defects located at the phase interfaces can be described by solitons, in terms of the kink-antikink pairs governed by the ${\varphi }^{4}$ model. The topological excitations in our theoretical model stem from the existent degeneracy of the two opposite ground states, inward and outward polarizations, of the unit cell. For the finite size soliton-lattice, we can define the topological charge $Q=(1/2a){\int }_{-L}^{L}{\partial }_{x}\theta {\rm{d}}x$ as the difference in the number of kinks and antikinks^34^. In our case, $Q=0$, which indicates each kink is followed by an antikink. Similar phenomena also emerge in various nonlinear physical contexts such as mechanical transmission lines, conducting polymers, ferroelectric phase transitions and so on^10,11^.

### Intrinsic length scale of soliton-lattice

**Fig. 4:**
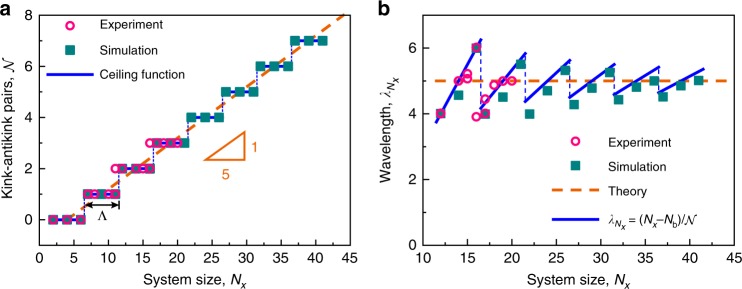
Size dependence of kink-antikink pairs and the soliton wavelength. **a** Number of kink-antikink pairs as a ceiling function of the system size. The step length is equal to the theoretical period $T$. The characteristic length to permit a stable kink-antikink pair is ${N}_{{\rm{c}}}=7$. Beyond this, a remarkable size-dependence arises. **b** Wavelength versus system size in experiments and simulations. The simulated behavior of a finite system gradually approaches that of the continuous model. Fitting the simulated data by ${\lambda }_{{N}_{x}}=({N}_{x}-{N}_{{\rm{b}}})/{\mathcal{N}}$ yields the depth of the boundary effects ${N}_{{\rm{b}}}=4$.

Investigating periodic soliton excitations within a definite-size system is, in fact, appealing for evident reasons: not only the finite-length situation is consistent well with the physical realities, but also by their intrinsic periodic features, finite-length soliton-lattices tend to reveal novel size-dependent properties strikingly distinct from those of the usual discrete or single solitons^13,14,39^. Consequently, we further explore the variation of the characteristics of the soliton-lattice with the number of unit cells ${N}_{x}$ in the metamaterial. Two intrinsic length scales are discovered (Fig. 4). When ${N}_{x}$ is less than a critical value ${N}_{{\rm{c}}}\approx 7$, uniform deformation takes place and no stable kink-antikink pairs emerge due to the boundary effects (see Supplementary Fig. 5 and Movie 2). With increasing system size ${N}_{x}$, the boundary effects diminish and the static soliton topology becomes admissible. Moreover, the number of kink-antikink pairs ${\mathcal{N}}$ varies with ${N}_{x}$: an increase in the length by $5$ leads to one more kink-antikink pair formation (Fig. 4a). Therefore, ${\mathcal{N}}$ is related to ${N}_{x}$ by the ceiling function ${\mathcal{N}}=\lceil ({N}_{x}-{N}_{{\rm{c}}})/\Lambda \rceil$, with step length $\Lambda =5$. Interestingly, the step length is the same as the theoretical period $T$, another intrinsic length scale in the metamaterial. In addition, the kink-antikink pairs are stably separated from each other with a proper spacing denoted by the wavelength ${\lambda }_{{N}_{x}}$ (see Supplementary Fig. 5), due to the interactions among the solitons themselves as well as those between the solitons and the free boundaries^10^. The wavelength likewise varies with the system size, following a twisted saw-tooth-wave function where ${\lambda }_{{N}_{x}}$ locally ramps upward with ${N}_{x}$ and then sharply drops (Fig. 4b). For small systems, i.e., ${N}_{x} \, < \, 4T$, the wavelength ${\lambda }_{{N}_{x}}$ shows a larger scatter, suggesting the ranges that system size matters. However, the scatter gets smaller and ${\lambda }_{{N}_{x}}$ gradually approaches the theoretical period $T$ of the continuous model as ${N}_{x}$ increases. This discontinuous size-dependence in ${\lambda }_{{N}_{x}}$ essentially originates from the existent intrinsic length scale $T$. Fitting the experimental and simulated results by an empirical formula ${\lambda }_{{N}_{x}}=({N}_{x}-{N}_{{\rm{b}}})/{\mathcal{N}}$, we get ${N}_{{\rm{b}}}=4$, which represents the size of the boundary regimes in present conditions. Remarkably, the observed size-dependent properties purport two intrinsic length scales: the critical system size ${N}_{{\rm{c}}}$ and the soliton-lattice period $T$, which are naturally associated with the architectural details. ${N}_{{\rm{c}}}$, related to ${N}_{{\rm{b}}}$, represents the smallest system size to robustly generate a kink-antikink pair, which quantitatively reflects the boundary effects and even the Saint-Venant principle^40^. $T$ is actually an characteristic length scale perpendicular to the compressive loadings and is distinct from the bulk solids^3,41^ and previously reported metamaterials^23,42^.

### General framework for programming solitons

**Fig. 5:**
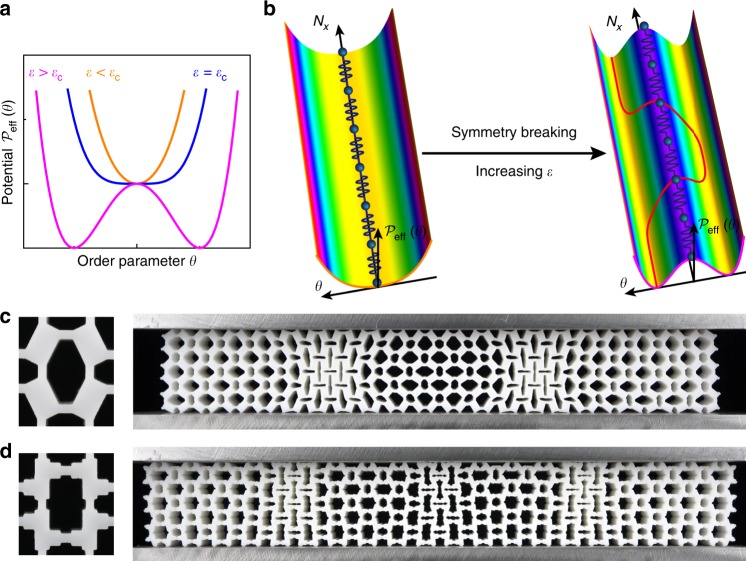
Programming periodic localizations in 2D metamaterials based on the general framework. **a**, **b** Illustrations of the evolution of the effective on-site potential with compressive strain, and the structural phase transition mechanism of the multistable systems. This general framework identifies the mechanism and provides the guideline for exciting static periodic solitons in metamaterials. **c**, **d** Unit cells and periodic localizations underlying the static kink/antikink excitations in Block-Spring and Rod-Spring metamaterials, respectively. The deformed configurations are shown at a compressive strain $\varepsilon =9.5 \%$. The architectures of the unit cells, and comparisons among the experimental, numerical and theoretical results of these metamaterials are shown in detail in the Supplementary Figs. 10–12.

The framework for excitation of static topological soliton-lattice is general, and not limited to the specific configuration shown in Fig. 1. The key lies in the quantifying competitions between the interaction energies and the effective on-site potentials of the unit cell, which further determines the phase transition type (Supplementary Note 3). The underlying physical mechanism revealed by the system provides a general framework for designing and programming ordered localizations in 2D metamaterials. Firstly, one need tailor a unit cell with multi-stability perpendicular to the loading direction, e.g., the stiffnesses satisfying $K{H}_{0}^{2} \, > \, 8({C}_{1}+{C}_{2})$ in the system given in Fig. 1 (also see Supplementary Fig. 9). Beyond a critical compressive loading strain ${\varepsilon }_{{\rm{c}}}$, its on-site potential reduces to degenerated states to trigger the bifurcation modes of the unit cell (Fig. 5a). Secondly, to excite the periodic localizations, strong interaction conditions with respect to the effective on-site potential barrier (illustrated in Fig. 5b) are required to ensure displacive phase transition, e.g., $4{C}_{{\rm{s}}}{d}^{2}\gg K{H}_{0}^{2}(1-\varepsilon )\varepsilon -2({C}_{1}+{C}_{2})$ for the case considered. To illustrate the generality of this framework, we fabricated and tested three more 2D metamaterials with different microstructures (i.e., the Block-Spring, Rod-Spring and Elliptic-Circular metamaterials shown in Fig. 5 and Supplementary Note 3). Their architecture and material details can be found in the Methods and Supplementary Note 3. As expected, static alternating kink/antikinks and thus periodic localizations are excited in these materials, and excellent agreement among the experimental, simulated and theoretical results was achieved (see Fig. 5c, d and Supplementary Figs. 10–12). The corresponding intrinsic length scales $T$ and ${N}_{{\rm{c}}}$ in the metamaterials (Fig. 5 and Supplementary Figs. 10–12) could also be programmed by the unit cell geometries.

## Discussion

Taken together, these theoretical, experimental, and numerical results show that the localized deformation of a perfect bi-stable cellular mechanical metamaterial under quasi-static compression can be highly ordered, in contrast to widely known homogeneous configurations or non-deterministic deformation-localization in solids. Our theoretical model reveals that, upon compression, the competition between the effective on-site potentials and the strong interaction energies of the constituent cells in the cellular metamaterial results in a displacive phase transition and a symmetry order reduction beyond a critical load. Accompanied by spontaneous symmetry breaking, static periodic kink-antikink pairs (i.e., topological solitons) are excited in the metamaterial, inducing an orderly localized configuration. A discrete size-dependence of the wavelength of the periodic solitons caused by an intrinsic length scale is also unveiled. We emphasize that the double-well potential and the strong coupling energy are pivotal factors that ensure the displacive phase transition and, evidently, can be programmed into the unit cell geometries to prescribe soliton-lattice configurations.

The striking size-dependent property of kink-antikink pairs, denoted by a ceiling function in our metamaterial systems, applies across multistable systems including molecular transport in restricted spaces^43^, atomic-scale frictions^16^, and domain switching^44^. This holds potential to be leveraged for a range of new metamaterials with programmable localized deformations for fields such as smart molecular robotics. Besides, the alternative kinks and antikinks count the reversals of the topological mechanical polarizations along the quasi-1D metamaterial chain. By encoding the inward and outward domain-states as mechanical bits which can be identified by probing the local order parameters, we envisage such robust reversal features are promising for enabling information storage and retrieval on static soliton-lattices^45^. More broadly, the results enable design of unit cell topologies with multistable states that can pave novel pathways to prescribing ordered localization excitations and controllable geometric phase transitions across a range of physical contexts.

## Methods

### Sample preparation

We fabricated our samples via a double-molding process. Firstly, we cast polyurethane solution into a 3D printed mold (mold I, analogous to the experimental model shown in Fig. 1a) to generate a structure consisting of arrays of elliptical cylinders (used as mold II). Secondly, we poured Hei-Cast 8000 polyurethane solution into the mold II and cured it at room temperature for 12 h. Release agent was sprayed onto the mold surfaces in advance to facilitate separation during the replication processes. After carefully removing the elliptical cylinders, a soft cellular metamaterial was produced. The out-of-plane thickness of the sample was $D$ = 70 mm, and the initial in-plane size was ${N}_{x}\times {N}_{y}=20\, \times 3$ (see Fig. 1), with ${N}_{x}$ and ${N}_{x}$ referring to the numbers of unit cells in the $x$ and $y$ directions, respectively. The semi-axes of the orthogonally oriented elliptical holes within the unit cell considered in Fig. 1 were chosen as ${b}_{1}=2{a}_{1}$= 6 mm and ${a}_{2}=2{b}_{2}=$ 3 mm. The cell neck thickness of $t=$ 1.5 mm was adopted to ensure easy processing. The dimensions of the unit cell can be written as ${L}_{x}=2({a}_{1}+{a}_{2}+t)$ and ${L}_{y}=2({b}_{1}+{b}_{2}+t)$.

### Experimental tests

Six samples were loaded under uniaxial compression using a Zwick/Roell testing machine (Zwick-Roell, Ulm, Germany). Before testing, sample surfaces and loading platens were covered with white Vaseline to reduce friction. Tests were conducted at a constant nominal strain rate of $3.1\times 1{0}^{-5}\ {{\rm{s}}}^{-1}$ to mimic quasi-static conditions. We verified that these loading rates represented quasistatic conditions by repeating tests on each sample at a rate of $1.0 \, \times 1{0}^{-4}\ {{\rm{s}}}^{-1}$ and obtaining nominally identical results. The height ${Y}_{0}={N}_{y}{L}_{y}$ and cross-section area $D{N}_{x}{L}_{x}$ of the structure were used for calculating the engineering strain and nominal stress, respectively. To study the size-dependent properties of the kink-antikink pairs, we fabricated samples with system size ${N}_{x}=20$ and performed initial uniaxial tests. After these initial tests, several columns of the samples were removed to obtain a smaller sample that was tested under conditions identical to the initial uniaxial test. This was repeated with increasingly smaller specimens to obtain results for size dependence (Fig. 4, Supplementary Fig. 5). Note that the because samples were made of an elastomeric material, loading history is not expected to have major effects on the stress-strain curves and deformation patterns, as confirmed by the tests. We have also marked necks of unit cells in the middle row of the sample (see Supplementary Fig. 2). A high resolution digital camera (SONY FDR-AX40, Tokyo, Japan) was used to record the positions of markers and the deformed configurations of the specimens as loading progressed (Supplementary Movies 1 and 2). This allowed us to measure the characteristic rotations $\theta$ of the unit cell and identify the deformed configurations as a function of the displacement ${u}_{y}$ (see Supplementary Fig. 2).

### Numerical simulations

The commercial finite element software Abaqus/Standard (Dassault Systmes, Vlizy-Villacoublay, France) was employed in our static finite element simulations. Based on the uniaxial testing results, the Mooney-Rivlin hyperelastic constitutive model was adopted to characterize the metamaterial. Material constants were taken as ${C}_{10}=$ 0.284 MPa and ${C}_{01}$= 0.432 MPa (Young’s modulus $E=$ 4.30 MPa and Poisson’s ratio $\nu =0.499$, see Supplementary Fig. 1) according to our experimental measurement. Plane strain, 4-node elements with a hybrid formulation and reduced integration were used. Mesh sensitivity analyses showed that about $1 \, \times 1{0}^{4}$ elements per unit cell were adequate to ensure convergent numerical results.

*Simulation of full samples*: we conducted nonlinear FE simulations on the full metamaterial, sandwiched between two rigid plates, with frictionless contact between the sample and the plates (Supplementary Fig. 2). Quasistatic compressive displacement was applied to the rigid plates and an artificial inter-dissipation factor of $1\times 1{0}^{-4}$ was specified to stabilize the simulations. Self-contact of the holes was ignored in the modeling, because only moderate compression prior to full densification was considered. The reaction force $F$ and the relative displacement ${u}_{y}$ between the two rigid plates were used to calculate the macroscopic nominal stress and engineering strain via $\sigma =F/(D{N}_{x}{L}_{x})$ and $\varepsilon ={u}_{y}/({N}_{y}{L}_{y})$. In addition, the characteristic angle of the unit cell in the deformed metamaterial was defined as the relative rotations of the midpoint pairs on the vertical necks (see Supplementary Fig. 2).

*On-site potential of the unit cell*: two static steps were employed to obtain the on-site potential of the unit cell. First, a static compressive displacement, i.e. ${u}_{y}=2\delta$, was applied to the unit cell, and the resulting characteristic angle and strain energy of the unit cell, denoted by ${\theta }_{0}$ and ${U}_{{\rm{cell}}}({\theta }_{0})$ were obtained. Second, relative rotation loads on the four irregular rods were imposed with the vertical compression fixed. In this way, the strain energy ${U}_{{\rm{cell}}}(\theta )$ versus rotation $\theta$ curve could be extracted. The on-site potential of the unit cell was then defined as ${{\mathcal{P}}}_{{\rm{cell}}}(\theta ) \,\, \triangleq \,\, {U}_{{\rm{cell}}}(\theta )-{U}_{{\rm{cell}}}({\theta }_{0})$, which is dependent on the compressive load and structural stiffness (see Supplementary Note 3 for details).

*Hinge stiffness*: the hinge stiffness of the simplified model is dependent on the geometry of the elliptical holes and neck thickness^30,46^. In the ideal case such as necks of constant cross-section, or circular holes with the slender limit $t/{a}_{1}\ll 1$, analytical expressions for hinge stiffness versus the geometry and bulk material constants can be obtained. However, the necks for the samples considered in this study are of finite size and have variable cross-sections (see Supplementary Fig. 3). Therefore, analytical expressions for the hinge stiffness are hard to attain and FE simulations were conducted to extract them. The hinges are represented by linear springs with stiffnesses obtained by calculating the linear responses to three different loadings (i.e., tension, shear, and bending as shown in the inset of Supplementary Fig. 3). To apply tension, shear and bending, we coupled the motions of the nodes on the cross-sectional plane of each dissected part with that of a virtual node, which is displaced by the corresponding displacement or rotation. We used the reaction loads associated with these virtual node displacements and rotations to calculate the tension, shear and bending stiffnesses by $K=F/\delta$, ${C}_{{\rm{s}}}=F/u$ and ${C}_{i}=M/{\theta }_{i}(i=1,2)$, respectively.

### Verification of the general framework

The general framework for predicting solitons in metamaterials was verified by considering a range of classes of metamaterials. For all of these (see Fig. 5 and Supplementary Figs. 10–12), sample preparation, experiments and simulations proceeded according to the above procedures (cf. Fig. 1). Material constants, geometrical parameters and other experimental/numerical details can be found in the Supplementary Note 3.

## Supplementary information

Supplementary Information

Supplementary Movie 1

Supplementary Movie 2

Description of Additional Supplementary Files

## Data Availability

The data that support the plots within this paper and other findings of this study are available from the corresponding author upon request.
