# Supplementary material for: Programmable and robust static topological solitons in mechanical metamaterials
Source: Nat Commun. 2019 Dec 6;10:5605. doi: 10.1038/s41467-019-13546-y (PMC6898320; doi:10.1038/s41467-019-13546-y)
Supplement: Supplementary file 1 — Supplementary Information [file 41467_2019_13546_MOESM1_ESM.pdf]

Supplementary Information of "Programmable and robust static topological solitons in mechanical metamaterials"

Yafei Zhang[1], Bo Li[1], Q. S. Zheng[1], Guy M. Genin[2,3] & C. Q. Chen[1,*]

[1]*Department of Engineering Mechanics, CNMM and AML, Tsinghua University, Beijing, 100084, P.R. China*

[2]*Mechanical Engineering and Materials Science, Washington University, St. Louis, MO 63130, USA*

[3]*NSF Science and Technology Center for Engineering Mechanobiology, St. Louis, MO 63130, USA*

**Correspondence to: C. Q. Chen (chencq@tsinghua.edu.cn)*

This Supplementary Information provides further details of the main text and is organized as follows, Supplementary Note 1: Details of finite element (FE) simulations; Supplementary Note 2: Size effects and defect insensitivity; Supplementary Note 3: General static soliton framework; and Supplementary Movies.

Supplementary Note 1: Details of finite element (FE) simulations

Material constitutive model

The solid material constituting the metamaterial is an incompressible hyperelastic elastomer. In the FE modeling, its constitutive behavior is assumed to follow the Mooney-Rivlin hyperelastic model, with the strain energy density given by[1]

$$W = C_{10}(\bar{I}_1 - 3) + C_{01}(\bar{I}_2 - 3) \tag{S1}$$

where $\bar{I}_1$ and $\bar{I}_2$ are the first and second invariant of $\mathbf{B}$= $(\det\mathbf{B})^{-1/3}\mathbf{B}$ with $\mathbf{B} = \mathbf{F} \cdot \mathbf{F}^{\mathrm{T}}$ being the left Cauchy-Green tensor of the deformation gradient $\mathbf{F}$. The corresponding Cauchy stress $\boldsymbol{\sigma}$ can be written as

$$\boldsymbol{\sigma} = -\frac{2}{3}(C_{10}\bar{I}_1 - C_{01}\bar{I}_2)\mathbf{I} + 2C_{10}\mathbf{B} - 2C_{01}\mathbf{B}^{-1}. \tag{S2}$$

Under uniaxial tension, Eq. (S2) reduces to

$$\sigma_{11}^{\mathrm{nor}}/(\alpha - \alpha^{-2}) = (2C_{10} + 2C_{01}\alpha^{-1}) \tag{S3}$$

where $\sigma_{11}^{\mathrm{nor}}$ is the nominal uniaxial tensile stress and α is the stretch in the loading direction. By fitting Eq. (S3) against the experimental results under uniaxial tension (see Supplementary Fig. 1), the material constants $C_{10} = 0.284\,\mathrm{MPa}$ and $C_{01} = 0.432\,\mathrm{MPa}$ (Young's modulus $E = 6(C_{01} + C_{10}) = 4.30\,\mathrm{MPa}$) are obtained.

FE simulation and spring stiffness

The experimental and simulated responses of the metamaterial to uniaxial compression are represented in Supplementary Fig. 2a, showing excellent agreement with each other. Note that, to stabilize the simulations, an artificial inter-dissipation factor of 1×10^{-4} is introduced in the FE modeling. This specific choice is made by trial and error: Much more or less dissipation would result in non-realistic deformed patterns or unstable simulations, respectively.

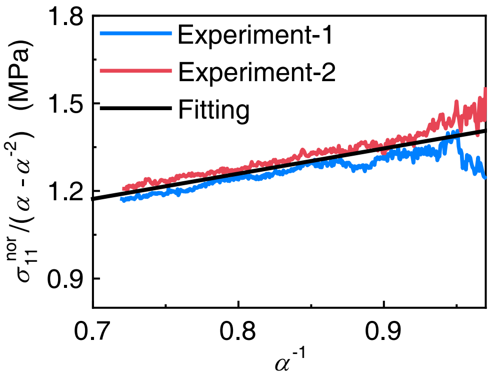

Supplementary Figure 1. Experimental determination of the material constants. Curve fitting of the tension testing results of the incompressible hyperelastic elastomer specimens by Eq. (S3) gives $C_{10} = 0.284\,\text{MPa}$ and $C_{01} = 0.432\,\text{MPa}$.

The nominal stress versus engineering strain curves ($\sigma \sim \varepsilon$) indicate instability occur during loading, which is confirmed by the sharp drop in σ beyond the threshold strain ε_c (see Supplementary Fig. 2b). To capture the symmetry breaking and the formation of periodic solitons in the metamaterial, the characteristic rotation angle of the n-th unit cell in the middle row is defined as $\theta_n \triangleq (\theta_{n,l} - \theta_{n,r})/2$, with $\theta_{n,l} = \arctan\left((l_x^{\rm u} - l_x^{\rm d})/(l_y^{\rm u} - l_y^{\rm d})\right)$ and $\theta_{n,r} = \arctan\left((r_x^{\rm u} - r_x^{\rm d})/(r_y^{\rm u} - r_y^{\rm d})\right)$ being the rotations of the lift and right midpoint pairs (i.e., $l^{\rm u} \sim l^{\rm d}$ and $r^{\rm u} \sim r^{\rm d}$) marked on the vertical necks (Supplementary Fig. 2c), respectively. In the experiments, we use a digital camera (SONY-FDR-AX40) to record the deformation process. The rotation angles of the midpoint pairs can be calculated by employing the image processing technology via MATLAB(R2017b, The MathWorks Inc.). Similarly, in the simulation, the coordinates of the midpoint pairs can be extracted, and the corresponding rotation angles can be obtained. We can further introduce the notion of polarization to the metamaterial consisting of bi-stable unit cells, by harnessing the outward and inward polarizations to the deformed unit cell with $\theta_n > 0$ and $\theta_n < 0$, respectively. Accordingly, the characteristic rotation angle θ depicts the states of the unit cells and, in fact, serves as the order parameter during the structural phase transition.

Based on the experiments and simulations, as shown in Supplementary Fig. 2, the deformations of the metamaterial can be characterized by the stretching of the plates, and the bending and shearing of the necks in the unit cells. As the ligament thickness t is significantly small, the deformation of the metamaterial is mainly concentrated in the neck region[2,3]. We

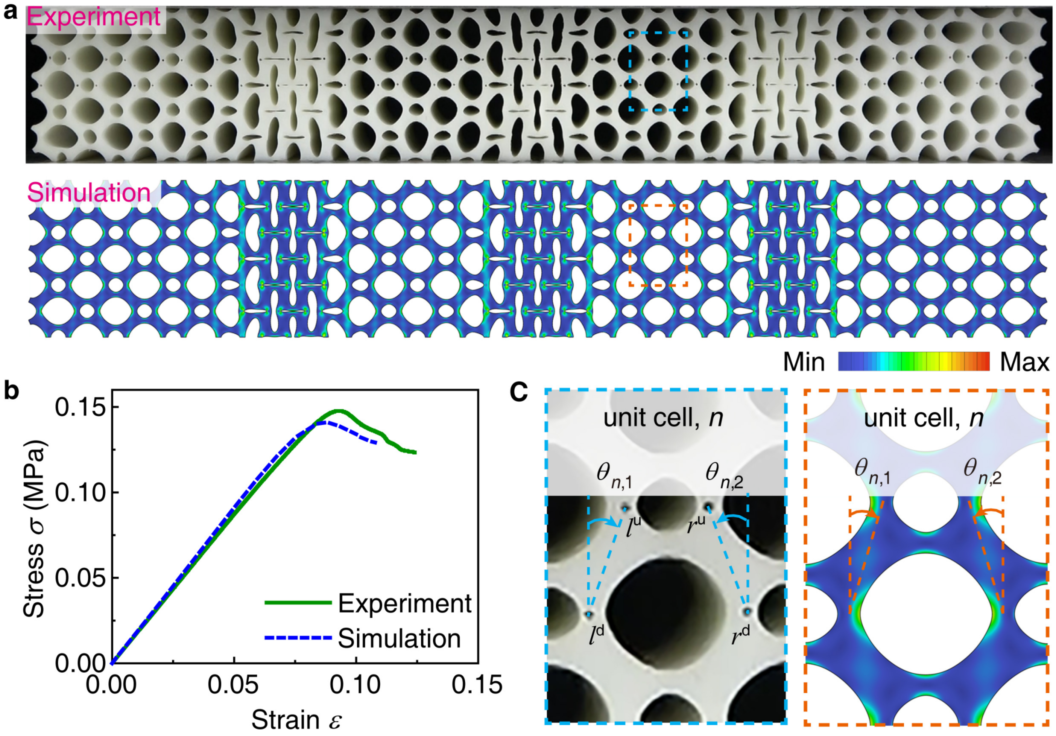

Supplementary Figure 2. Experimental and simulation results on the responses of the metamaterial to uniaxial compression. **a** Deformed configurations at global compressive strain of $\varepsilon = 10.7\%$ and the simulated result also shows the von Mises stress filed of the metamaterial. **b** Nominal stress versus engineering strain curves ($\sigma \sim \varepsilon$), and **c** Detailed deformations of the n-th unit cell, with the characteristic rotation defined by $\theta_n \triangleq (\theta_{n,l} - \theta_{n,r})/2$. The FE simulations are first validated against the experiments and are then expanded to calculate the spring stiffness and analyze the size effects of soliton-lattice.

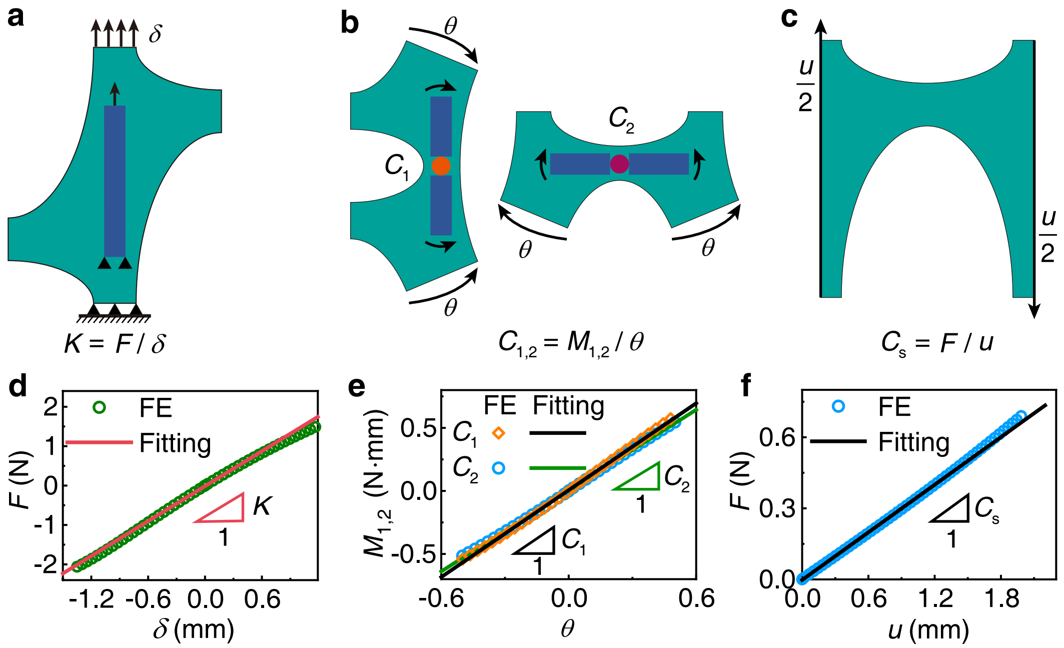

Supplementary Figure 3. FE simulations for determining the spring stiffness. **a-c** The FE model to calculate the stiffness of tension K, torsional $C_i (i = 1, 2)$ and shear springs C_s, respectively, with the equivalent rod-spring elements and boundary conditions graphed on them. **d-e** The reaction force/moment versus applied loads curves. The stiffness can be obtained by a linear fitting of the FE results within our considered range of strains, and in present cases, $K = 1.478\,\mathrm{N \cdot mm^{-1}}$, $C_1 = 1.150\,\mathrm{N \cdot mm}$, $C_2 = 1.069\,\mathrm{N \cdot mm}$ and $C_\mathrm{s} = 0.3230\,\mathrm{N \cdot mm^{-1}}$, respectively.

develop rod-spring elements to represent the deformation features (Supplementary Fig. 3a-c). The corresponding stretching, torsional and shearing stiffness are defined as $K = F/\delta$, $C_{1,2} = M_{1,2}/\theta$ and $C_\mathrm{s} = F/u$, respectively. Since analytical solutions for these parameters are unavailable due to the irregular neck geometries, they can be calculated by FE simulations. In the simulation, plane strain elements (i.e., 4-nodes with hybrid formulation and reduced integration) are employed. The load and boundary conditions are illustrated in Supplementary Fig. 3a-c and fittings are conducted for the FE results within the considered ranges of strain (Supplementary Fig. 3d-f). Note that these stiffnesses are dependent on the geometries of the elliptical holes. The dependency can be quantified by FE simulations, as detailed in our recent work[3].

Supplementary Note 2: Size effects and defect insensitivity

We perform FE simulations to investigate the effects of vertical system size N_y on the soliton-lattice excitations, where $N_x = 8, 20, 30$ and 39 are respectively specified. The soliton-lattice patterns of models with $N_x = 20$ while $N_y = 1, 2, 3$ and 5 are shown in Supplementary Fig. 4. Our results suggest that the localizations are almost independent of N_y, yet the upper bound is still an open question when the slenderness ratios N_y/N_x of metamaterials cannot be ignored. However, the geometric feature of the long strip structures (i.e., $N_y \ll N_x$) permits us to construct a 1D model to study the intrinsic localization behavior.

We further investigate the effects of system size N_x on the excitations of kink-antikink pairs by experiments and simulations. The number of kink-antikink pairs and their spacing show striking size-dependent features with varying N_x and $N_y = 3$ being fixed (Figure 4 in main text). The experimental samples with $N_x = 19, 18, ..., 4$ are fabricated by gradually cutting off some columns of unit cells from the initial samples with $N_x = 20$. The models with $N_x = 1, 2, ..., 42$ are mainly investigated by the FE simulations, and the larger size, i.e., $N_x = 72$ is also studied to further confirm our theoretical predictions. Soliton-lattice patterns with $N_x = 4, 8, 15$ and 18 are presented in Supplementary Fig. 5. Evidently, our simulations agree well with the experiments.

We also consider the role of initial mirror symmetry in the excitations of static soliton-

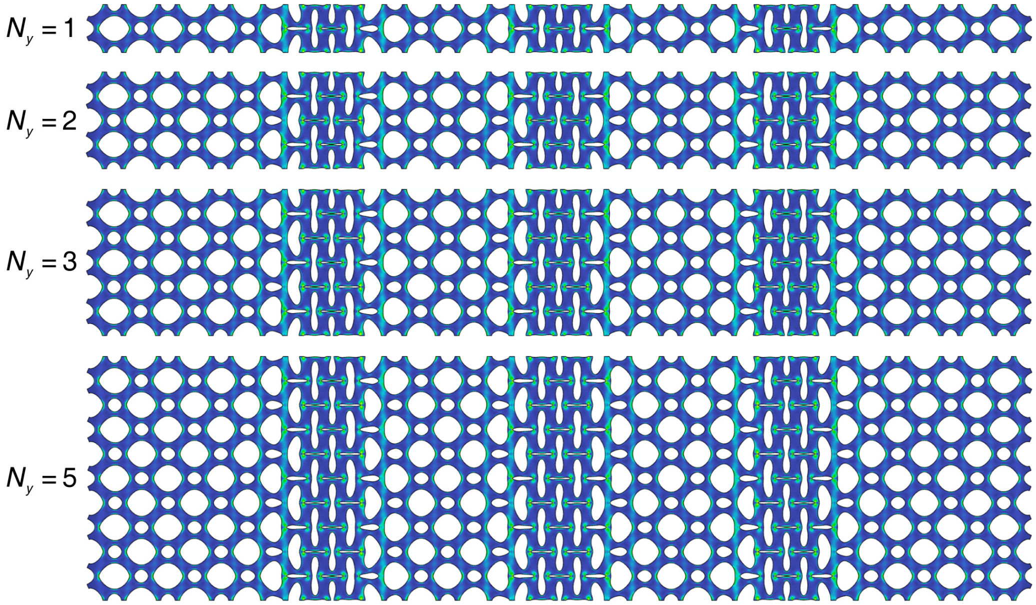

Supplementary Figure 4. Soliton-lattice pattern are insensitive to the vertical size N_y to some extent. The system size in the x direction $N_x = 20$ is specified, while that in the y direction $N_y = 1, 2, ..., 5$ are respectively investigated. The deformed configurations are selected at the vertical engineering strain of $\varepsilon = 10.7\%$.

lattice. Evidently, The deformed pattern displayed in our main text maintains the initial reflection symmetry, even though the translational symmetry of the unit cell in the x direction is broken. We break the reflection symmetry in x direction of the metamaterial by cutting the left boundary cells in the middle row of the FE model $N_x \times N_y = 20 \times 3$. Our simulation results show that these three kink-antikink pairs robustly emerge until about 5 unit cells are removed (see Supplementary Fig. 6). Notably, the kink-antikink pair near the defect is just slightly affected if we remove only one unit cells, due to the Saint-Venant principle. However, the positions of the disturbed kink-antikink pairs gradually move to the right side (away from the defect) and the reflection symmetry of the whole model in x direction is obviously broken.

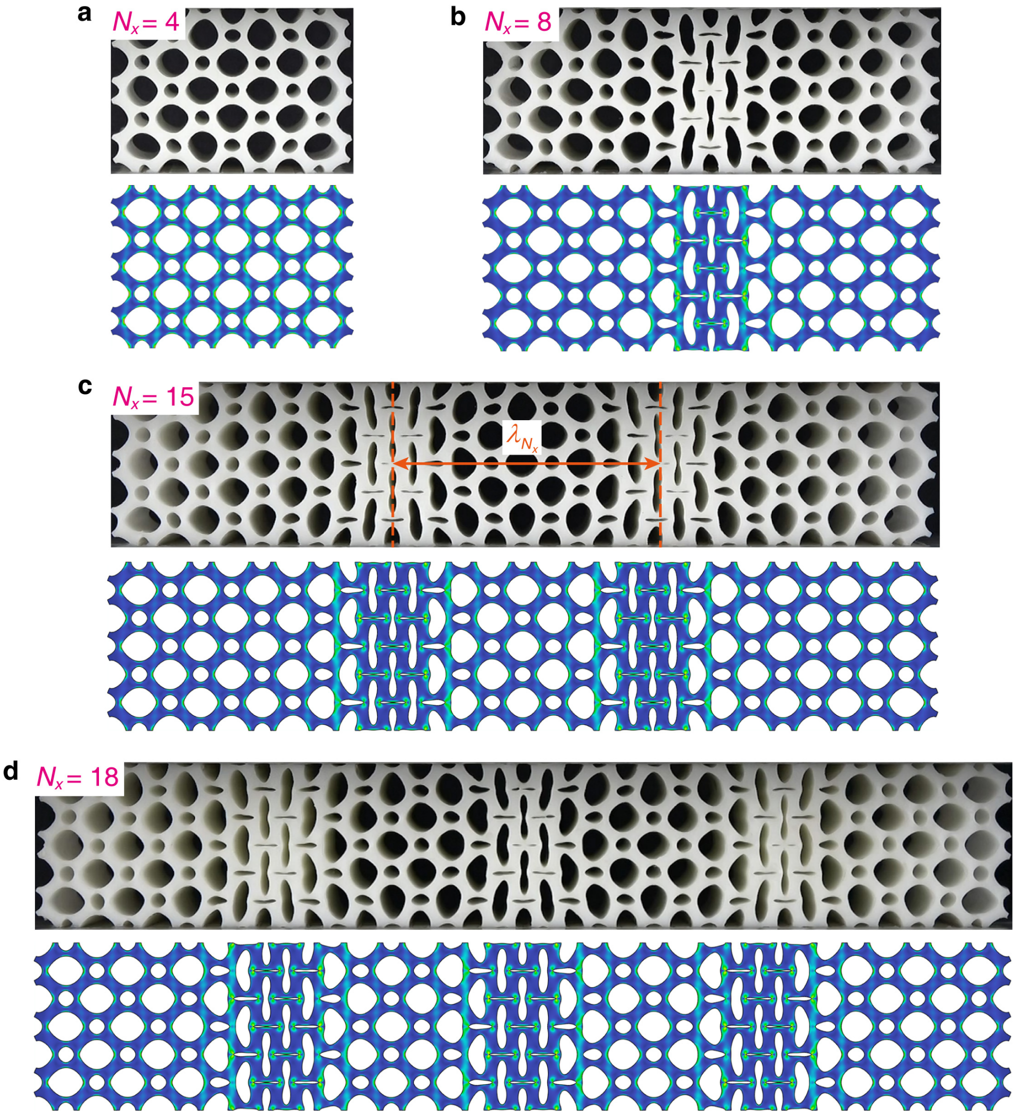

Supplementary Figure 5. $\boldsymbol{N_x}$**-dependent properties of kink-antikink pairs**. Typical experimental and simulated results of kink-antikink pairs in soliton-lattices with the length of $N_x = 4, 8, 15$ and 18, respectively. The soliton wavelength λ_{N_x}, illustrated in **c**, is defined by the spacing of kink-antikink pairs normalized by the width of the unit cell L_x. The selected strain of the results is $\varepsilon = 10.7\%$.

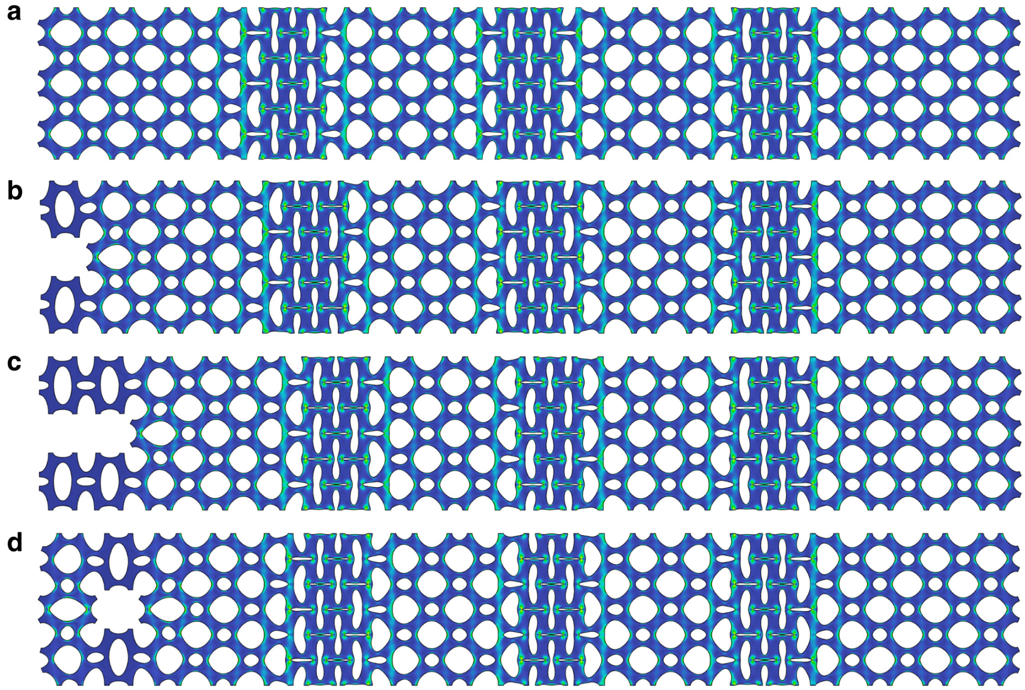

Supplementary Figure 6. The effects of reflection symmetry breaking on the static soliton excitations. **a** The deformed pattern with no structural defects. **b-d** Deformed configurations of the metamaterials with initial reflection symmetry broken by removing the unit cells $(N_x, N_y) = (1, 2)$,$(1\&2, 3)$ and $(2, 3)$, respectively. There pairs of kink-antikinks robustly emerge even though some unit cells are cut off, among which the left and middle pairs are slightly driven to the right one.

Supplementary Note 3: General static soliton framework

On site potential of the unit cell

We calculate the on-site potentials of the unit cell via both numerical and theoretical procedures. In our simulations, two static loading steps are conducted. First, a uniform compressive displacement $u_y = 2\delta$ is applied to the unit cell (Supplementary Fig. 7a), which results in the deformed configuration shown in Supplementary Fig. 7b. We denote the corresponding rotation angle and strain energy by θ_0 and $U^{\mathrm{FE}}_{\mathrm{cell}}(\theta_0)$, respectively. Secondly, we keep the compressive load and twist the four plates to change the rotation angle (consider the first compressive state as the initial configuration (Supplementary Fig. 7b)). During the torsion, the current configuration (Supplementary Fig. 7c), the corresponding rotation angle θ and strain energy $U^{\mathrm{FE}}_{\mathrm{cell}}(\theta)$ can be recorded and extracted via Abaqus/standard software. Setting the configuration with θ_0 as the zero potential state, the on-site potentials of the unit cell can be, therefore, approximately calculated by $\mathcal{P}^{\mathrm{FE}}_{\mathrm{cell}}(\theta) = U^{\mathrm{FE}}_{\mathrm{cell}}(\theta) - U^{\mathrm{FE}}_{\mathrm{cell}}(\theta_0)$.

Based on the thin ligament assumption mentioned in the main text, we propose an effective rod-spring model to capture the deformation feature of the unit cell (Fig.2 in main text and Supplementary Fig. 7a). It consists of elastic rods connected by neck springs (torsional and shearing springs) whose stiffness can be calculated by FE simulations (Supplementary Fig. 3). As t/a_1 is small enough, the bending of the rods can be ignored. Under the compression $u_y = 2\delta$, the strain energy of the unit cell is resulted from the stretching of the rods, torsion and/or shear of the necks springs. Ignoring the asymmetric modes of the unit cell, the energy function can be written as

$$U_{\mathrm{cell}}(\Delta H; \theta) = 2(2C_1 + C_2)\theta^2 + 2K(\Delta H)^2 \tag{S4}$$

where $\Delta H = H_0 - H = H_0 - (H_0 - \delta)\sec\theta$. As $\theta \ll 1$, taking the approximation $\sec\theta = 1 + \theta^2/2 + 5\theta^4/24 + o(\theta^4)$, Eq. (S4) can be written as

$$U_{\mathrm{cell}}(\delta; \theta) = U_e(\theta^2 - \Theta_{\mathrm{cell}})^2 + D_1 \tag{S5}$$

with

$$U_e = \frac{1}{6}K(3H_0 - 8\delta)(H_0 - \delta), \tag{S6}$$

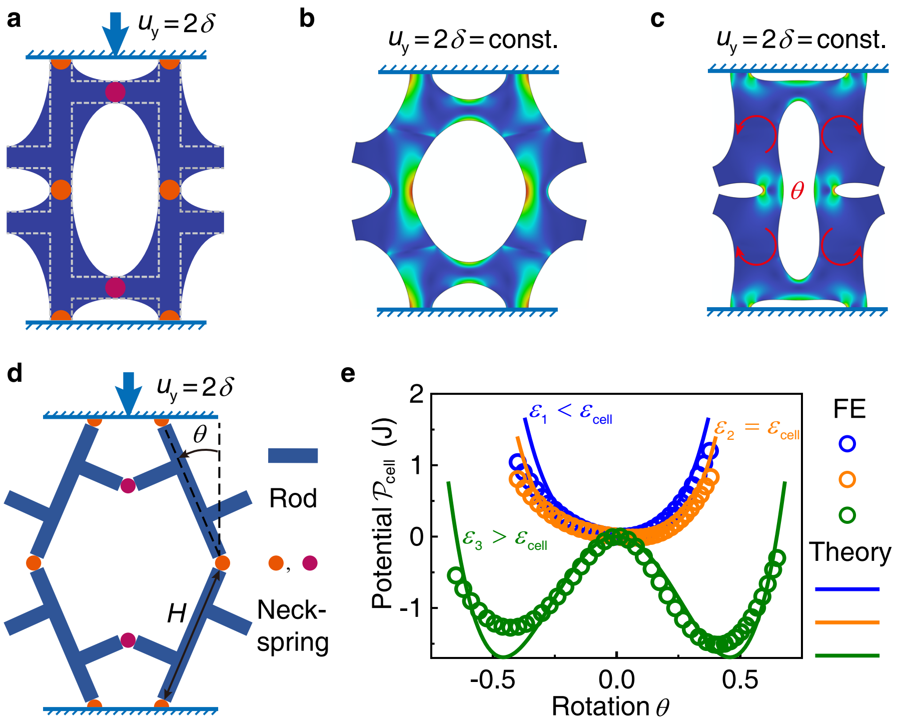

Supplementary Figure 7. Procedures to calculate the on-site potentials of the unit cell. **a** The undeformed configuration of the FE and effective rod-spring model of the unit cell. **b** The deformed configuration of the unit cell in the first loading step: under the vertical compression $u_y = 2\delta = 1.92\,\mathrm{mm}$. **c** A selected current state of the unit cell in the second loading step: under the torsional load applied to the configuration in **b**. **d** A geometry-allowed configuration of the effective rod-spring model with rotation angle θ and rod length H. The neck springs are denoted by dots. **e** Numerical and theoretical on-site potentials of the unit cell at three strains $\varepsilon_1 = 1.11\%$, $\varepsilon_2 = 2.89\%$ and $\varepsilon_3 = 10.7\%$.

$$\Theta_{\text{cell}} = \frac{6\left(K\delta(H_0-\delta)-(2C_1+C_2)\right)}{K(3H_0-8\delta)(H_0-\delta)} \tag{S7}$$

and the θ-independent constant $D_1 = 2K\delta^2 - \Theta_{\text{cell}}\left(K\delta(H_0-\delta)-(2C_1+C_2)\right)$. In fact, there are numerous geometry-allowed configurations (various θ as shown in Supplementary Fig. 7d) of the rod-spring model under the compression $u_y = 2\delta$. However, the actual states (i.e., $\theta = \theta_0$) should be determined by minimizing U_{cell} via $\partial U_{\text{cell}}/\partial\theta\,|_{\theta=\theta_0} = 0$. Therefore, the deformations of the unit cell can be regarded as a 'quasiparticle' sited in a potential filed, seeking and occupying the stable state. Analogous to the definition of numerical on-site potential via the simulation procedure, this theoretical on-site potential of the unit cell can be defined by

$$\mathcal{P}_{\text{cell}}(\delta;\theta) = U_{\text{cell}}(\delta;\theta) - D_1. \tag{S8}$$

Deformed configurations of the unit cell are related to the features of $\mathcal{P}_{\text{cell}}$. As $\Theta_{\text{cell}} \leqslant 0$, $\mathcal{P}_{\text{cell}}(\delta;\theta)$ only has one stable state $\theta = 0$, which indicates a compression deformation. On the contrary, $\Theta_{\text{cell}} > 0$ or equivalently $KH_0^2 > 4(2C_1+C_2)$ and $3H_0/8 > \delta > \delta_{\text{cell}} = \left(H_0 - \sqrt{H_0^2 - 4\left(2C_1+C_2\right)/K}\right)\Big/2$, $\mathcal{P}_{\text{cell}}(\delta;\theta)$ has two stable states at $\theta = \mp\sqrt{\Theta_{\text{cell}}}$, which corresponds to the inward and outward polarizations, respectively. The numerical and theoretical on-site potentials are presented in Supplementary Fig. 7e, showing good agreement with each other to some extent. It should be noted that $\varepsilon \triangleq \delta/H_0$ and in this Note, $\theta = 0$ is selected as the zero potential state to facilitate the comparisons. Setting $\Theta_{\text{cell}} = 0$, we obtain the critical strain $\varepsilon_{\text{cell}} = \delta_{\text{cell}}/H_0$, above which the on-site potential will be nonconvex.

Structural phase transition and static periodic-soliton

To uncover the physical mechanism of the excitations of static periodic solitons in the metamaterials, a quasi-1D rod-spring model system is constructed (Fig. 3a in the main text and Supplementary Fig. 8a). We consider a finite size model consists of $N_x \times 1$ unit cells, as illustrated in Supplementary Fig. 8a. The unit cells are connected by bending and shearing springs (magenta dots in Fig. 3a and Supplementary Fig. 8a). The vertical compressive load $u_y = 2\delta$ is uniformly applied at the top boundary of the rod-spring model system.

Deformation. Here we retain the assumption $t/a_1 \ll 1$ to obtain analytic solutions. Therefore, stretching of the rods and, bending and shear of the necks dominate the deformation of this quasi-1D rod-spring model system. We further notice that only bending occurs at the yellow neck due to the symmetry in the vertical direction, while bending and shear are

coupled at the magenta necks owing to the incompatible deformations of adjacent unit cells. These three kinds of deformations at the necks are governed by bending stiffness $C_i (i = 1, 2)$ and shear stiffness C_s, respectively.

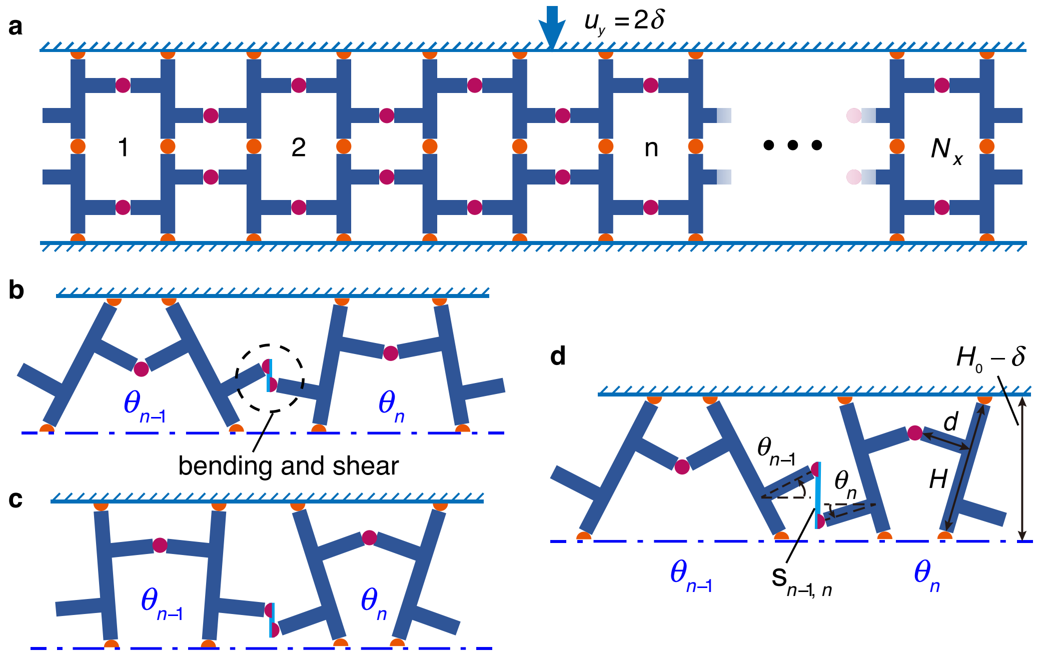

Supplementary Figure 8. Schematic of the rod-spring model system and typical deformations. **a** The 1D rod-spring model system of the metamaterial with N_x unit cells. **b-d** Three typical deformed configurations of the unit cells within which the asymmetric modes are ignored. Only a half of the unit cells are depicted due to the symmetry and each unit cell has a characteristic rotation angle $\theta_n (n = 1, 2, ..., N_x)$. The length of the blue line $s_{n-1,n}$ denotes the shear deformation between the $(n-1)$-th and n-th unit cells.

Klein-Gordon equation and $\boldsymbol{\lambda\varphi^4}$ theory. For simplicity, we tacitly ignore the asymmetric modes of the unit cell and, therefore, a characteristic rotation angle $\theta_n (n = 1, 2, ..., N_x)$ can be specified to the n-th unit cell, as defined in Supplementary Fig. 2. Based on this assumption, three typical deformed configurations in the rod-spring model system under the compression $u_y = 2\delta$ are listed in Supplementary Fig. 8b-d. The total strain energy of the system is related to these deformations by

$$E_\mathrm{sys} = \sum_n \left(\frac{1}{2} C_\mathrm{s} s_{n-1,n}^2 \cdot 2 + \frac{1}{2} \left(C_1 + C_2 \right) \theta_n^2 \cdot 8 + \frac{1}{2} K \left(\varDelta H \right)^2 \cdot 4 \right) \tag{S9}$$

where the first term works as the interactions between the adjacent $(n-1)$-th and n-th unit cells, and the last two terms result from the bending and stretching of the n-th unit cell

itself. It is noted that this strain energy E_{sys} is dependent on the numerous geometry-allowed configuration of the rod-spring system. However, the actual stable states will be found at stationary points of E_{sys}. Analogous to the analysis in Supplementary Fig. 7, we can regard the unit cells as quasiparticles and introduce a particle-chain model placed in the discrete potential fields to equilibrate the loading process (Fig. 3b in main text). The Lagrangian of the chain system can be written as

$$\mathcal{L} = \sum \left(E_{\text{inter}} + U_{\text{eff}}\right) = \sum_{n} \left(C_{\text{s}} s_{n-1,n}^{2} + 2C_2\theta_n^2 + U_{\text{cell}}\left(\theta_n\right)\right) \tag{S10}$$

where the interaction energy $E_{\text{inter}} \triangleq C_{\text{s}} s_{n-1,n}^{2} = C_{\text{s}} d^2(\sin\theta_{n-1} - \sin\theta_n)^2$ and the strain energy of the unit cell

$$U_{\text{eff}} \triangleq 2C_2\theta_n^2 + U_{\text{cell}}\left(\theta_n\right) = U_e(\theta_n^2 - \Theta_{\text{eff}})^2 + D_2 \tag{S11}$$

with

$$\Theta_{\text{eff}} = \left(K\delta(H_0 - \delta) - 2(C_1 + C_2)\right)/U_e \tag{S12}$$

and $D_2 = 2K\delta^2 - \Theta_{\text{cell}}\left(K\delta(H_0 - \delta) - 2(C_1 + C_2)\right)$. Note that the constant D_2 is θ-independent and can be omitted in the following derivations. Therefore, the effective on-site potential of the particle-chain model can be defined as $\mathcal{P}_{\text{eff}}\left(\theta_n\right) \triangleq U_{\text{eff}}\left(\theta_n\right) - D_2$.

Considering the Taylor expansions $\sin\theta = \theta - \theta^3/6 + o(\theta^4)$ as $\theta \ll 1$, the equilibrium equation can be found by $\partial\mathcal{L}/\partial\theta = 0$, that is,

$$-2C_{\text{s}} d^2(\theta_{n-1} + \theta_{n+1} - 2\theta_n) + \partial\mathcal{P}_{\text{eff}}\left(\theta_n\right)/\partial\theta_n = 0. \tag{S13}$$

The deformations of the chain model are governed by the features of $\mathcal{P}_{\text{eff}}\left(\theta_n\right)$: as $\Theta_{\text{eff}} < 0$, $\mathcal{P}_{\text{eff}}\left(\theta_n\right)$ only has one stable state and the system undergoes uniform deformations under compression; On the contrary, $\Theta_{\text{eff}} > 0$, or equivalently $KH_0^2 > 8(C_1 + C_2)$ and $3H_0/8 > \delta > \delta_{\text{s}} = \left(H_0 - \sqrt{H_0^2 - 8\left(C_1 + C_2\right)/K}\right)\Big/2$ (based on Eq. (S12)). Hence, $\mathcal{P}_{\text{eff}}\left(\theta_n\right)$ is a double well potential which indicates the structural phase transitions are allowable in the metamaterials. The critical load $\delta_{\text{s}} = \delta\mid_{\Theta_{\text{eff}}=0}$ can be obtained by the losing of convex in $\mathcal{P}_{\text{eff}}\left(\theta_n\right)$. We develop a phase diagram to identify whether the unit cells of the metamaterials can possess monostable or bi-stable on-site potentials. Here, we are interested in the bi-stable phase (Green region in Supplementary Fig. 9a). Note that the effective on-site potential $\mathcal{P}_{\text{eff}}$ of unit cells in this region is evidently dependent on the loads. Gradually increasing the load beyond the threshold δ_{s}, $\mathcal{P}_{\text{eff}}$ varies form monostable to bi-stable (Supplementary Fig. 9b).

As $\delta > \delta_s$, we can expect two limiting physical regimes based on Eq. (S13): the order-disorder and displacive phase transitions, which essentially depend on the relative magnitudes of the coupling energy and the on-site potential-barrier height.

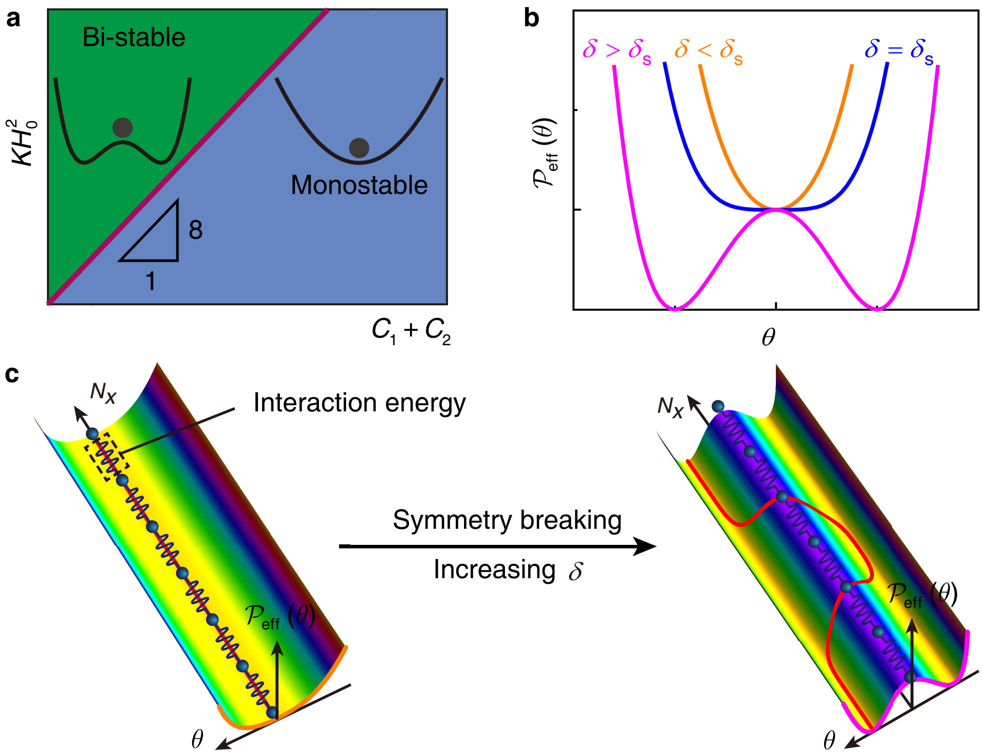

Supplementary Figure 9. Mechanism of displacive phase transitions in the metamaterials. a Phase diagram of the metamaterials with effective monostable and bi-stable states. **b** Schematic of effect on-site potentials of the metamaterials under different strain stages. The on-site potential exhibit bi-stable features as $\delta > \delta_s$. **c** Particle-chain model to schematically illustrate the displacive phase transition accompanied by the symmetry-breaking in the metamaterials, which induces the formation of the static periodic-solitons.

- *Order-disorder phase transitions.* As $2C_s d^2\left(2\sqrt{\Theta_{\mathrm{eff}}}\right)^2\Big/2 \ll U_e\Theta_{\mathrm{eff}}^2$ or equivalently $C_s d^2 \ll (K(H_0-\delta)\delta - 2(C_1+C_2))/4$ one has a collection of weakly coupled nonlinear oscillators, randomly displaced to $\theta \approx \pm\sqrt{\Theta_{\mathrm{eff}}}$. In this situation, the order-disorder phase transition takes place and induces random localizations in the metamaterials.
- *Displacive phase transitions.* In contrast, as $C_s d^2 \gg (K(H_0-\delta)\delta - 2(C_1+C_2))/4$

rotation angle θ changes slowly and the standard continuum approximations

$$\begin{aligned} &\theta_n \to \theta\,(x = n\ell) \\ &\theta_{n\pm1} \to \theta\,(n\ell) \pm \ell\partial_x\theta\,(n\ell) + \ell^2\partial_{xx}\theta\,(n\ell)/2 \end{aligned} \tag{S14}$$

can be used to reduce Eq. (S13) to

$$\partial_{xx}\theta - \partial_\theta V\,(\theta) = 0 \tag{S15}$$

where $V\,(\theta) = \lambda\,(\theta^2 - \Theta_{\text{eff}})^2 \Big/ 4$ and $\lambda = 2U_e/(C_{\text{s}}d^2\ell^2)$. Equation (S15) is the well-known nonlinear Klein-Gordon equation in the structural phase transition (or the $\lambda\varphi^4$ equation in gauge filed theory)[4]. It permits the topological periodic-soliton solution:[5,6]

$$\theta = a \cdot \text{sn}\left[b\sqrt{\lambda/2}x; m\right] \tag{S16}$$

where $\text{sn}\left[b\sqrt{\lambda/2}x; m\right]$ is the Jacobi elliptic function, $a = \sqrt{2m/(1+m)\Theta_{\text{eff}}}$ and $b = \sqrt{2/(1+m)\Theta_{\text{eff}}}$. The parameter m can be determined by the boundary conditions, and in present case $m = 0.47$. As to a finite system, the periodically localized configuration is usually termed as a soliton-lattice with kink width $w = \sqrt{(1+m)/(\lambda\Theta_{\text{eff}})}$. The normalized period is $T = 4\text{K}[m]/(b\ell\sqrt{\lambda/2})$ with $\text{K}[m]$ being the elliptic integral of the first kind.

- *Other trivial or more complex excitations.* In addition to the two limiting regimes, the trivial excitations such as $\theta_n = \pm\sqrt{\Theta_{\text{eff}}}$ are also the solution of Eq. (S13). Evidently, it is the lowest-energy state of the system because all unit cells (quasiparticles) silently reside at the bottoms of a potential well. The present displacive regime reported in the main text is, in fact, one kind of low-energy yet intrinsically nonlinear excitations above the lowest-energy state. Other more complex nonlinear excitations in the multistable metamaterial systems may also be expected, which is still an open question.

General soliton framework to program periodic localizations.

Remarkably, the structure phase transition mechanism revealed in this study, in fact, can serve as a general framework to excite static periodic-solitons and thereby program ordered localizations in mechanical metamaterials. Designing unit cells with multi-stabilities, for example, satisfying condition of $KH_0^2 > 8(C_1 + C_2)$ and $C_{\text{s}}d^2 \gg (K(H_0 - \delta)\delta - 2(C_1 +$

$C_2))/4$, the compressive load can trigger the displacive phase transitions in the metamaterials (see Fig. 5a,b in main text and Supplementary Fig. 9). Based on Eq. (S15), gradually increasing δ beyond the threshold δ_s, the effective on-site potential $\mathcal{P}_{\text{eff}}$ switches from monostable to bi-stable, and correspondingly, the deformation of the metamaterial undergoes a transition from uniform compression to ordered localization. This displacive phase transition accompanied by the spontaneous symmetry-breaking of the characteristic rotation (order parameter θ) further induces the excitations of static kink, as schematically illustrated in Supplementary Fig. 9b,c. The topological constraint of the double well potential indicates each kink should necessarily be followed by an antikink in our metamaterial systems. Our experiments and simulations clearly demonstrate this evolution process (see Fig. 1 in main text, Supplementary Fig. 2 and Movie 1).

To demonstrate the general utility of the static soliton framework for programming program periodic localizations, and to show that excitations of static soliton-lattices are not limited in a specific structure considered above, we used the framework to design and analyze three other classes of mechanical metamaterials (Supplementary Figs. 10-12) different from the metamaterial considered in Fig.1.

In the first class, the axial ratio of the orthogonally oriented elliptic holes was changed and the shape of the hole in our new models can be elliptic and circular (Elliptic-Circular metamaterial in Supplementary Figs. 12). In the other two cases, the metamaterials were constructed by block and rod structures (Block-Spring and Rod-Spring metamaterial in Supplementary Figs. 10 and 11, respectively). The geometry parameters of the unit cells are depicted in detail in Supplementary Figs. 10a-12a.

In our experiments, we fabricate 4 samples for each kind of metamaterial, and get the repeatable deformed patterns at the extremely low strain ratio $2.5 \times 10^{-5}\text{s}^{-1}$ (see Supplementary Figs. 10-12). There are 2, 3 and 2 kink-antikink pairs of robustly emerging in the Elliptic-Circular (EC), Rod-Spring (RS) and Block-Spring (BS) metamaterials, respectively, after the displacive phase transition of the metamaterials (Supplementary Figs. 10d-12d). Finite element simulations are also conducted via the Abaqus/standard software, and they faithfully reproduce the deformation details of the experimental results (Supplementary Figs. 10e-12e). To stabilize the simulation, trial and error procedure mentioned in Supplementary Note 1 is also employed during the FE modeling and the inter-dissipation

factor introduced here is with the magnitude $10^{-5} \sim 10^{-6}$. Note that all the deformed configurations in Supplementary Figs. 10-12 are selected at the strain of $\varepsilon = 0.095$, and the slightly higher critical stress/strain in the experiments compared to that in simulations (see Supplementary Figs. 10b-12b) is due to the inner damping of the real rubber materials[7].

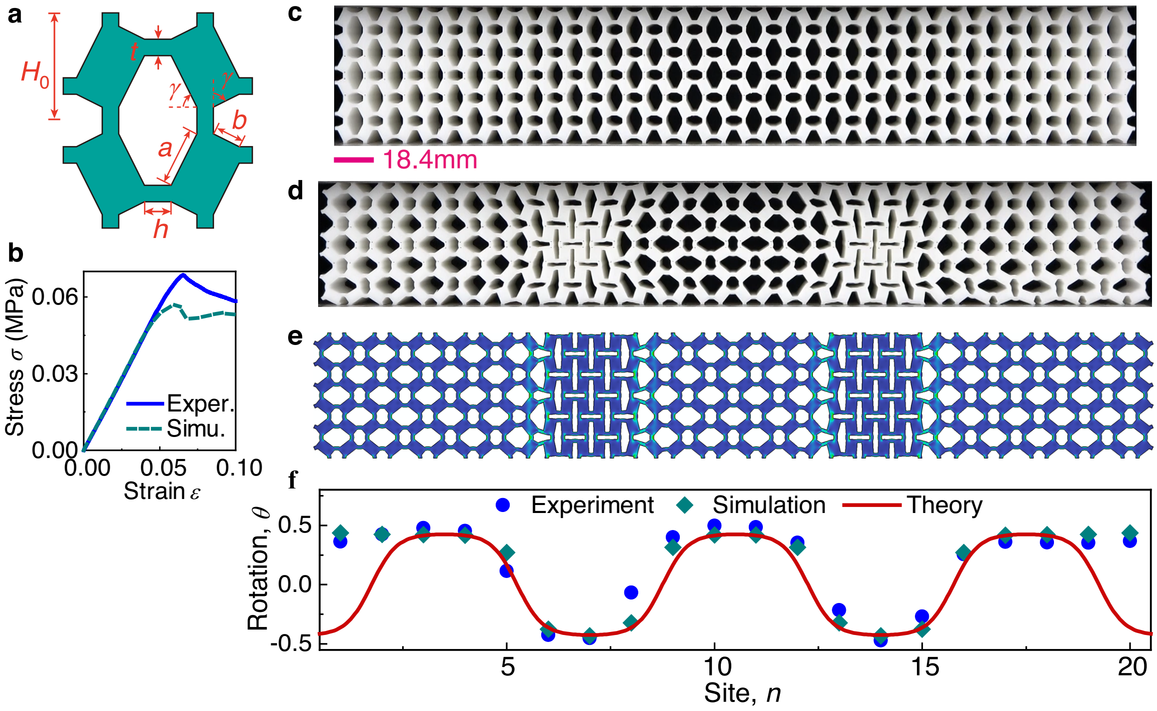

Supplementary Figure 10. Architectural details of the unit cell and results of the Block-Spring metamaterial. **a** Unit cell of the Block-Spring (BS) metamaterial with the geometric parameters: $a = 2b = 5.6$ mm, inclination angle $\gamma = \arctan(b/a)$, neck thickness $t = 1.6$ mm and neck length $t = 2.6$ mm. **b** Experimental and simulated nominal stress versus strain curve of the BS metamaterial. **c** The initial configuration of the BS metamaterial with $N_x \times N_y = 20 \times 3$ and thickness $D = 50$ mm. **d,e** Deformed configurations of the BS metamaterial in the experiment and simulation at the strain of $\varepsilon = 9.5\%$, where two pairs of kink-antikinks emerge in this metamaterial. **f** Experimental and simulated rotation angles (extracted from models in **d** and **e**) confirm the theoretical prediction of the general framework, and the period $T \simeq 7$.

The constitutive material of the samples is still characterized by the Mooney-Rivlin hyperelastic model, and the corresponding material constants and spring stiffness of the theoretical model are list in Supplementary Table 1. Evidently, the spring stiffness is dependent on the architectures of the unit cells and can be programmed into the theoretical

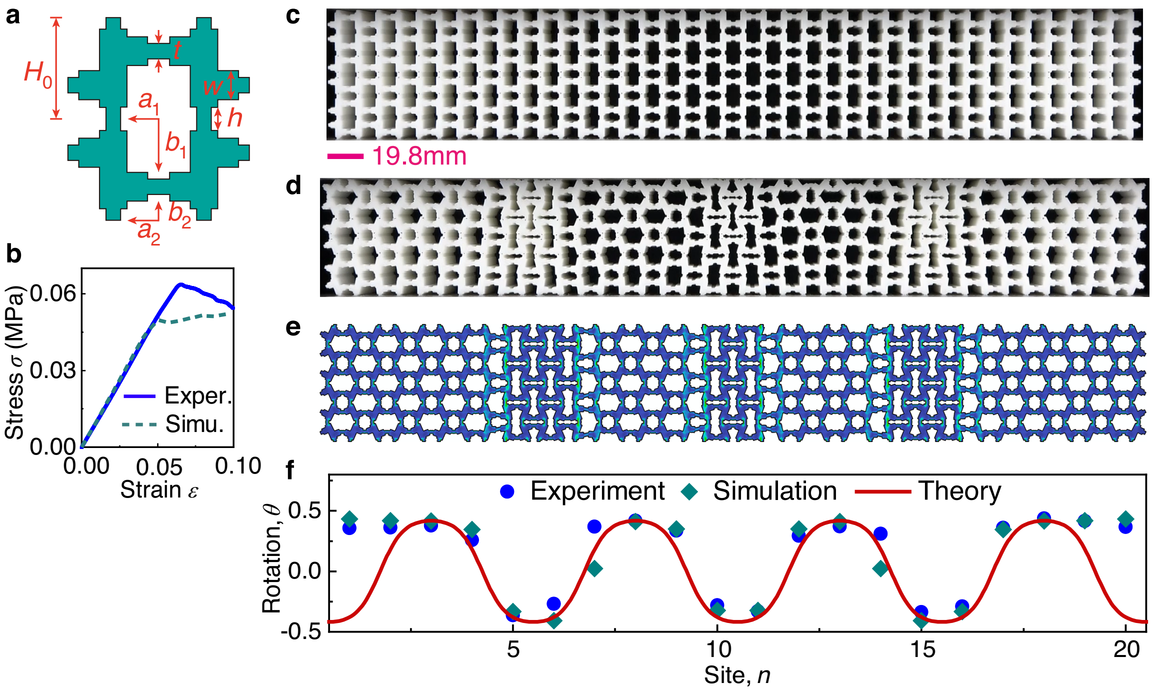

Supplementary Figure 11. Architectural details of the unit cell and results of the Rod-Spring metamaterial. **a** Unit cell of the Rod-Spring (RS) metamaterial with the geometric parameters: $a_1 = a_2 = 3.45\,\mathrm{mm}$, $b_1 = 5.5\,\mathrm{mm}$, $b_2 = 2.0\,\mathrm{mm}$, rod thickness $w = 3.0\,\mathrm{mm}$, neck thickness $t = 1.5\,\mathrm{mm}$ and neck length $h = 2.4\,\mathrm{mm}$. **b** Experimental and simulated nominal stress versus strain curve of the RS metamaterial. **c** The initial configuration of the RS metamaterial with $N_x \times N_y = 20 \times 3$ and thickness $D = 50\,\mathrm{mm}$. **d,e** Deformed configurations of the RS metamaterial in the experiment and simulation at the strain of $\varepsilon = 9.5\%$, where three pairs of kink-antikinks emerge in this metamaterial. **f** Experimental and simulated rotation angles (extracted from models in **d** and **e**) confirm the theoretical prediction of the general framework, and the period $T \simeq 5$.

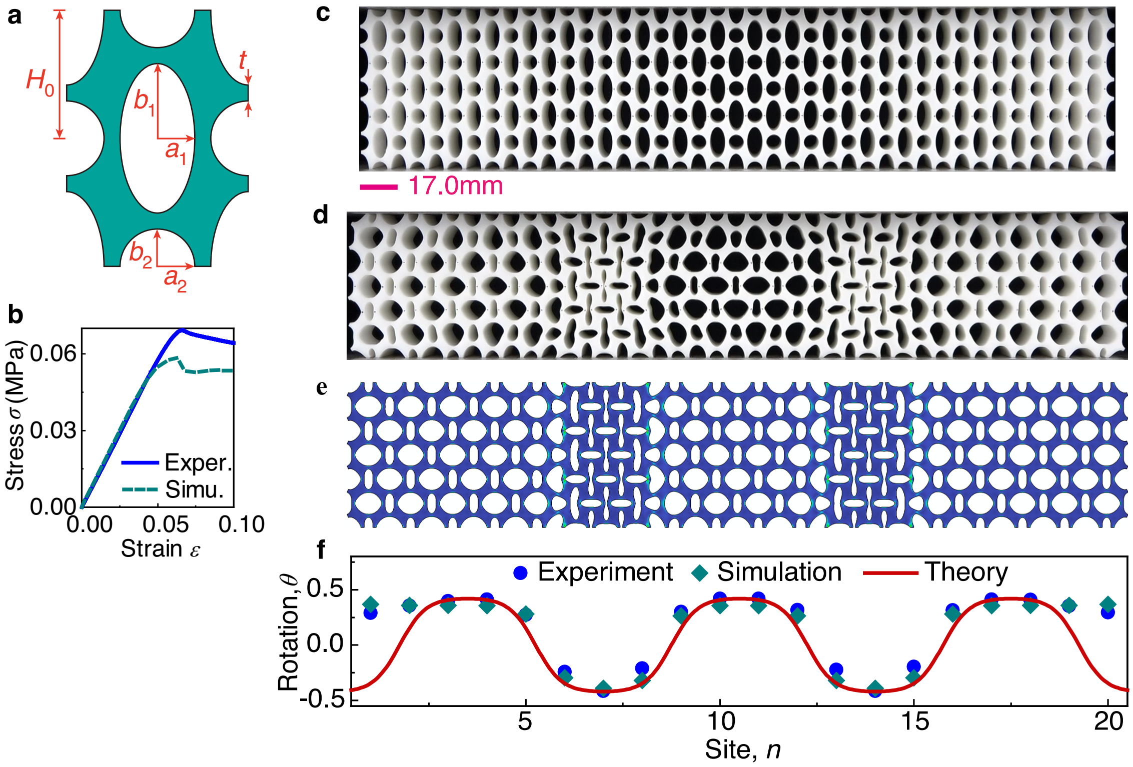

Supplementary Figure 12. Architectural details of the unit cell and results of the Elliptic-Circular metamaterial. **a** Unit cell of the Elliptic-Circular (EC) metamaterial with the geometric parameters: $a_1 = a_2 = b_2 = 3.5\,\text{mm}$, $b_1 = 2a_1 = 7.0\,\text{mm}$ and neck thickness $t = 1.5\,\text{mm}$. **b** Experimental and simulated nominal stress versus strain curve of the EC metamaterial. **c** The initial configuration of the EC metamaterial with $N_x \times N_y = 20 \times 3$ and thickness $D = 50\,\text{mm}$. **d,e** Deformed configurations of the EC metamaterial in the experiment and simulation at the strain of $\varepsilon = 9.5\%$, where two pairs of kink-antikinks emerge in this metamaterial. **f** Experimental and simulated rotation angles (extracted from models in **d** and **e**) confirm the theoretical prediction of the general framework, and the period $T \simeq 7$.

frameworks. Introducing the spring stiffness into Eq. (S15) and setting the Jacobi elliptic modulus $m = 0.9147, 0.8216$ and 0.9494 respectively, as expected, the theoretical results show excellent agreements with the experimental and simulated rotation angles shown in Supplementary Figs. 10f-12f, which confirms the general theoretical framework proposed in this work.

Supplementary Table 1. Materials constants and spring stiffness of the Block-Spring (BS), Rod-Spring (RS) and Elliptic-Circular (EC) metamaterials.

Models	C_{10}/MPa	C_{01}/MPa	$K/\mathrm{N}\cdot\mathrm{mm}^{-1}$	$C_1/\mathrm{N}\cdot\mathrm{mm}$	$C_2/\mathrm{N}\cdot\mathrm{mm}$	$C_\mathrm{s}/\mathrm{N}\cdot\mathrm{mm}^{-1}$
BS	0.1847	0.3255	1.0102	0.6546	0.6516	0.1213
RS	0.1935	0.3410	1.1344	0.6507	0.6321	0.1075
EC	0.1866	0.3289	0.8327	0.6055	0.8242	0.1875

Supplementary Movies

The structural phase transition and static soliton-lattice formation of the metamaterials during the quasi-static loading process are demonstrated in the movies. As presented in the main text and method, the samples is made from Mooney-Rivlin hyperelastic elastomer (material constants $C_{10} = 0.284\,\mathrm{MPa}$ and $C_{01} = 0.432\,\mathrm{MPa}$) with geometric parameters $a_1 = a_2 = 3\,\mathrm{mm}$, $b_1 = 4b_2 = 6\,\mathrm{mm}$ and $t = 1.5\,\mathrm{mm}$. The samples are lubricated by white Vaseline and undergo a sufficiently low strain rate of $3.1 \times 10^{-5}\mathrm{s}^{-1}$ to mimic the quasi-static loading. The movies, encoded in the form of H.246, were recorded and play back at 25 fps.

Supplementary Movie 1

Supplementary Movie 1 is about the structural phase transition and static soliton-lattice formation in the metamaterial with $N_x \times N_y = 20 \times 3$. Top panel: the metamaterial sample and loading device; Bottom panel: the characteristic rotation angle field of $N_x = 20$ unit cells in the x direction; Right panel: the nominal compressive stress versus engineering strain curve ($\sigma \sim \varepsilon$). Displacive phase transition takes place and static soliton-lattice emerges during the instability of the metamaterial (a sharp decrease of the $\sigma \sim \varepsilon$ curve). Playback

sped up ×546 and ×195 at the first 5 s and last 6 s of the movie, respectively.

Supplementary Movie 2

Supplementary Movie 2 is about the macro-homogeneous deformation of a metamaterial with $N_x \times N_y = 4 \times 3$. Top panel: the metamaterial sample and loading device; Bottom-lift panel: the characteristic rotation angle field of $N_x = 4$ unit cells in the x direction; Bottom-right panel: the nominal compressive stress versus engineering strain curve ($\sigma \sim \varepsilon$). No stable static kink is excited during the loading process due to the boundary effects (system size $N_x = 4 < N_c = 7$). Playback sped up ×360 in this movie.

Supplementary References

1. Rivlin, R. S. Large elastic deformations of isotropic materials. IV. Further developments of the general theory. *Phil. Trans. R. Soc. Lond. A* **241**, 379–397 (1948).
2. Coulais, C., Kettenis, C. & van Hecke, M. A characteristic length scale causes anomalous size effects and boundary programmability in mechanical metamaterials. *Nat. Phys.* **14**, 40–44 (2018).
3. Zhang, Y., Wang, Y. & Chen, C. Q. Ordered deformation localization in cellular mechanical metamaterials. *J. Mech. Phys. Solids* **123**, 28–40 (2019).
4. Dauxois, T. & Peyrard, M. *Physics of Solitons* (Cambridge University Press, Cambridge, 2006).
5. Cooper, F., Khare, A., Mihaila, B. & Saxena, A. Exact solitary wave solutions for a discrete $\lambda\phi^4$ field theory in $1+1$ dimensions. *Phys. Rev. E* **72**, 036605 (2005).
6. Dikandé, A. M. Bound states in one-dimensional Klein-Gordon systems admitting periodic-kink soliton excitations. *Phys. Scr.* **60**, 291–293 (1999).
7. Janbaz, S., McGuinness, M. & Zadpoor, A. A. Multimaterial control of instability in soft mechanical metamaterials. *Phys. Rev. Appl.* **9**, 064013 (2018).
