# Supplementary material for: Programmable and robust static topological solitons in mechanical metamaterials
Source: Nat Commun. 2019 Dec 6;10:5605. doi: 10.1038/s41467-019-13546-y (PMC6898320; doi:10.1038/s41467-019-13546-y)
Supplement: Supplementary file 4 — Description of Additional Supplementary Files [file 41467_2019_13546_MOESM4_ESM.docx]

**Title:** Supplementary Movie 1

**Description:** Uniaxial compression induced formation of static solitons. The size of the metamaterial is *N_x_*×*N_y_*=20×3. The upper panel shows the deformation evolution, with the corresponding site rotation and stress-strain curve given in the lower panel.

**Title:** Supplementary Movie 2

**Description:** Uniaxial compression induced uniform deformation. The size of the metamaterial is *N_x_*×*N_y_*=4×3. The upper panel shows the deformation evolution, with the corresponding site rotation and stress-strain curve given in the lower panel
